# Powerful large scale inference in high dimensional mediation analysis

**DOI:** 10.1371/journal.pcbi.1013880

**Published:** 2026-01-14

**Authors:** Asmita Roy, Xianyang Zhang

**Affiliations:** 1 Department of Biostatistics/Bloomberg School of Public Health, Johns Hopkins University, Baltimore, Maryland, United States of America; 2 Department of Statistics, Texas A&M University, College Station, Texas, United States of America; University of Virginia, UNITED STATES OF AMERICA

## Abstract

In genome-wide epigenetic studies, determining how exposures (e.g., Single Nucleotide Polymorphisms) affect outcomes (e.g., gene expression) through intermediate variables, such as DNA methylation, is a key challenge. Mediation analysis provides a framework to identify these causal pathways; however, testing for mediation effects involves a complex composite null hypothesis. Existing methods, such as Sobel’s test or the Max-P test, are often underpowered in this context because they rely on null distributions determined under only a subset of the null space and are not optimized for the multiple testing burden inherent in high-dimensional data. To address these limitations, we introduce MLFDR (Mediation Analysis using Local False Discovery Rates), a novel method for high-dimensional mediation analysis. MLFDR leverages local false discovery rates, calculated from the coefficients of structural equation models, to construct an optimal rejection region. We demonstrate theoretically and through simulation that MLFDR asymptotically controls the false discovery rate and achieves superior statistical power compared to recent high-dimensional mediation methods. In real data applications, MLFDR identified 20%–50% more significant mediators than existing methods, demonstrating its ability to uncover biological signals missed by conventional approaches.

## Author summary

The paper presents a novel approach to high-dimensional mediation analysis through a local false discovery rate (MLFDR) screening algorithm. It addresses the limitations of traditional methods like Sobel’s test and maxP, which are underpowered in high dimensional setting. We extend local FDR principles to composite null hypotheses, and derive a screening rule with a closed-form expression for false discovery proportion. We also show that MLFDR has comparable or better results than two recently-proposed methods, MDACT [30], HDMT [3] across a wide range of data types and models. We also provide a two-step global latent factor adjustment using surrogate variable analysis [9].

## 1 Introduction

Mediation analysis serves as a critical tool for deciphering the biological mechanisms underlying genetic associations with diseases identified in Genome-Wide Association Studies (GWAS). By bridging the gap between genetic variants and clinical outcomes, mediation analysis reveals intermediate pathways and elucidates causal relationships. As GWAS continues to uncover a vast number of genetic associations, translating these findings into actionable insights for precision medicine and therapeutic development becomes increasingly important. For instance, cigarette smoking is known to alter DNA methylation and gene expression [12]; concurrently, DNA methylation often regulates gene expression directly [4,14]. Investigating the mediating effect of DNA methylation on gene expression—particularly in the presence of environmental exposures like smoking—is therefore essential. However, these analyses are complicated by high-dimensional outcomes and clinical confounders, such as patient age, which influences both gene expression and DNA methylation heterogeneity [7,19]. This article addresses the statistical challenges inherent in such high-dimensional mediation problems.

Historically, [1] introduced the regression-based definition of mediation analysis, often referred to as the “product of coefficients method,” which examines the significance of the product of the exposure-mediator and mediator-outcome coefficients. More recently, the literature has expanded through the “counterfactual framework” [8,15,17,23–26], which provides a causal interpretation for natural direct and indirect effects across various models, including those with non-linearities and binary or survival outcomes.

Let *X* denote the exposure, *M*_*i*_ the *i*th mediator, and *Y* the outcome. Under the product of coefficients approach, mediation analysis tests the null hypothesis $H_{0,i}:\alpha_{i}\beta_{i}=0$, where $\alpha_{i}$ represents the effect of *X* on *M*_*i*_, and $\beta_{i}$ represents the effect of *M*_*i*_ on *Y*. This creates a composite null hypothesis comprising three distinct cases: (i) $\alpha_{i}=0,\beta_{i}\neq 0$; (ii) $\alpha_{i}\neq 0,\beta_{i}=0$; or (iii) $\alpha_{i}=0,\beta_{i}=0$. Assuming no unmeasured confounders, classical tests like Max-P [13] and Sobel’s test [18] are known to be conservative under case (iii), as statistical inference is typically derived from distributions determined by cases (i) and (ii). In genome-wide studies, however, the sparse nature of omics data implies that $\alpha_{i}=0$ and $\beta_{i}=0$ hold for the majority of markers. Recent methods such as JS-mixture (HDMT) [3] and DACT [11] attempt to address this by explicitly modeling the composite nature of the null. JS-mixture improves power by using a mixture-null distribution of maximum p-values, adapting [20]’s procedure to estimate component proportions. DACT estimates the proportions of null $\alpha_{i}$ and $\beta_{i}$ separately to combine case-specific p-values. However, [30] recently demonstrated that DACT suffers from False Discovery Rate (FDR) inflation under dense alternatives and proposed a modified version (MDACT) that computes the statistic’s distribution via numerical integration to improve p-value accuracy.

While JS-mixture and MDACT offer improvements over classical methods, they are not theoretically optimal regarding power. The FDR literature is broadly divided into p-value-based and local FDR-based rejection regions. Local FDR, a Bayesian approach, ranks hypotheses by the posterior probability that a case is null given the observed statistics; this ranking often differs from that based on p-values. [22] demonstrated that, except in cases of symmetric alternatives, local FDR and p-value-based orderings diverge. Furthermore, [22] proved that the local FDR-based oracle procedure is optimal: among all methods controlling the marginal FDR (mFDR), the local FDR approach yields the highest number of rejections. While the power advantage is negligible for symmetric alternatives, it becomes significant when the alternative distribution is asymmetric. Motivated by these theoretical properties, we propose MLFDR, a local FDR-based screening algorithm designed specifically for high-dimensional mediation analysis. Our contributions to the literature are as follows:

1. We extend the concept of local FDR to the composite null hypothesis setting, deriving a screening rule with a closed-form expression for the corresponding false discovery proportion (FDP).
2. We validate the method across a diverse array of data types—including continuous and binary variables, and scenarios with exposure-mediator interactions—demonstrating robust performance across various model specifications. We specifically incorporate Surrogate Variable Analysis (SVA) to adjust for latent confounding and illustrate the method’s efficacy in multiple mediator setups with univariate or clinical outcomes.
3. MLFDR offers optimal power improvement over existing methods while maintaining asymptotic FDR control. Extensive simulations confirm its superiority over MDACT and HDMT in terms of power and error rate control.
4. We provide theoretical guarantees for the identifiability and global optimality of our model under relatively mild assumptions, proving FDR control for both the oracle and adaptive procedures.

The remainder of this paper is organized as follows. Sect 2 contains the main results. Specifically, Sect 2.1 outlines the screening procedure for detecting significant mediators. Sect 2.2 presents simulation studies. Sect 2.3 discusses extensions of MLFDR to composite alternatives and latent factor models, which can account for unmeasured confounding and pleiotropy. Sect 3 provides an in-depth analysis of Prostate Cancer data and Lung cancer data from The Cancer Genome Atlas (TCGA), exploring SNP-CpG-gene expression pathways and causal pathways between smoking habits and gene expression, respectively. Sect 5 details the methodology. An R package implementing the method is available at https://github.com/asmita112358/MLFDR as well as in CRAN. Theorems proving the large-sample FDR control of MLFDR are provided in Section E of S1 Text.

Finally, we distinguish our approach from other recent efforts in high-dimensional mediation analysis. [33], [32], and [16] address the multiple mediator problem specifically for survival outcomes. Closer to our framework, [21] and [5] utilize local FDR-based rejection regions; the former approximates the alternative as a mixture of Gaussian distributions, while the latter constructs regions based on p-values. Our work advances this domain in two specific aspects: (i) we incorporate a general prior for the coefficients *α* and *β* to estimate the *exact* posterior density for computing the local FDR, rather than relying on approximations; and (ii) we offer theoretical guarantees for the local FDR estimates obtained via the EM algorithm, a property not previously established in this context.

## 2 Results

### 2.1 Method overview

**Fig 1:**
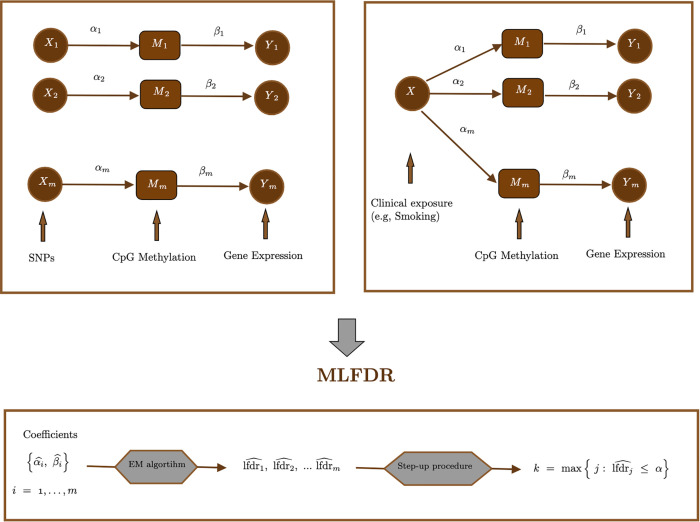
Schematic diagram of MLFDR.

This section outlines the workflow of MLFDR; a schematic representation of the framework is provided in Fig 1. Consider a study involving *n* independent samples. For each testing unit i=1,…,m, we observe an exposure variable *X*_*i*_, a mediator *M*_*i*_, and an outcome *Y*_*i*_. Biologically, these variables may represent distinct contexts: for example, *X* may denote a patient’s smoking history (shared across *i*), with $\{M_{i}{\}}_{i=1}^{m}$ representing CpG methylation sites and $\{Y_{i}{\}}_{i=1}^{m}$ representing gene expression levels. Alternatively, the analysis may focus on the functional impact of Single Nucleotide Polymorphisms (*X*_*i*_) on gene expression (*Y*_*i*_) as mediated by CpG methylation (*M*_*i*_) [3].

The mediation model posits that the exposure *X*_*i*_ influences the outcome *Y*_*i*_ through the intermediate variable *M*_*i*_, rather than solely through a direct relationship. We denote the coefficient for the exposure-mediator relationship ($X_{i}\to M_{i}$) as $\alpha_{i}$, and the coefficient for the mediator-outcome relationship ($M_{i}\to Y_{i}$) as $\beta_{i}$. In Fig 1, solid arrows indicate these direct effects.

We aim to test the composite null hypothesis against the alternative for each unit *i*:

$$H_{0,i}:\alpha_{i}\beta_{i}=0\;\text{versus}\;H_{1,i}:\alpha_{i}\beta_{i}\neq 0,\;i=1,2,\ldots ,m.$$ (1)

The composite null hypothesis *H*_0,*i*_ can be decomposed into three disjoint component nulls, $H_{0,i}=H_{00,i}\cup H_{01,i}\cup H_{10,i}$, defined as:

$$H_{00,i}:\alpha_{i}=0\text{ and }\beta_{i}=0,\;H_{10,i}:\alpha_{i}\neq 0\text{ and }\beta_{i}=0,\;H_{01,i}:\alpha_{i}=0\text{ and }\beta_{i}\neq 0,$$ (2)

for i=1,2,…,m.

We consider a mixture prior for $\left(\alpha_{i},\beta_{i}\right)$, where the probability of each disjoint component nulls $H_{00},H_{10}$ and *H*_01_ occur with probability $\pi_{00},\pi_{10}$ and $\pi_{01}$ respectively. The marginal prior distributions of $\alpha_{i}$ and $\beta_{i}$, respectively, are degenerate zero under the null and follow a normal prior with an unknown mean and variance under the alternative. The marginal distribution of the least squares coefficient estimates $\left\{{\hat{\alpha }}_{i},{\hat{\beta }}_{i}\right\}$ given the latent states is computed, and the unknown parameters including the null proportions are estimated using EM algorithm.

Using these estimates, we compute the local false discovery rate (local FDR) for each coefficient pair, denoted as ${\hat{\text{lfdr}}}_{i}$ for i=1,…,m. As the local FDR represents the posterior probability that the *i*-th hypothesis is null given the observed statistics, a lower value indicates stronger evidence against the null. Consequently, we define the rejection region for the composite null hypothesis as ${\hat{\text{lfdr}}}_{i}\leq \delta$. The threshold *δ* is determined adaptively using the step-up procedure proposed by [22]. The complete algorithm is detailed in the Methods section.

Additionally, we introduce an extended algorithm (MLFDR2) which can deal with scenarios where the marginal priors of $\alpha_{i}$ and $\beta_{i}$ follow mixture normal distributions under the alternative. A composite alternative leads to a joint distribution of $\left\{{\hat{\alpha }}_{i},{\hat{\beta }}_{i}\right\}$ with more than 4 mixture components, which can often be computationally burdensome. We introduce a two-step EM alogrithm that estimates the parameters of the marginal distributions of $\left\{{\hat{\alpha }}_{i}\right\}$ and $\left\{{\hat{\beta }}_{i}\right\}$ in the first step, then uses these estimates to run another EM algorithm that computes the probabilities of each mixture component.

We also discuss another extension using Surrogate Variable Analysis [10] which can account for unmeasured confounders and pleiotropy in the model. Details are presented in Sect 2.3.

### 2.2 Simulation studies

We evaluate the performance of MLFDR through extensive simulations under two distinct mixture proportion scenarios: a *dense* alternative and a *sparse* alternative. Following the setups in [3], the latent class probabilities are defined as:

- **Dense alternative:** $(\pi_{00},\pi_{10},\pi_{01},\pi_{11})=(0.4,0.2,0.2,0.2).$
- **Sparse alternative:** $(\pi_{00},\pi_{10},\pi_{01},\pi_{11})=(0.88,0.05,0.05,0.02).$

We consider sample sizes of n∈{100,300} and fix the number of mediators at *m* = 1000. The parameter controlling the signal strength of mediation, *τ*, varies from 0.1 to 1.9 in increments of 0.2. The non-zero coefficients are generated as $\alpha_{i}=0.05\tau +h_{i}$ (under *H*_10_ and *H*_11_) and $\beta_{i}=-0.5\tau +g_{i}$ (under *H*_01_ and *H*_11_), where the noise terms follow $h_{i}∼N(0,1/n)$ and $g_{i}∼N(0,4/n)$.

We compare the empirical FDR and power of MLFDR against two competing methods: MDACT [30] and HDMT (JS-mixture) [3]. The simulation settings are detailed below.

**Fig 2:**
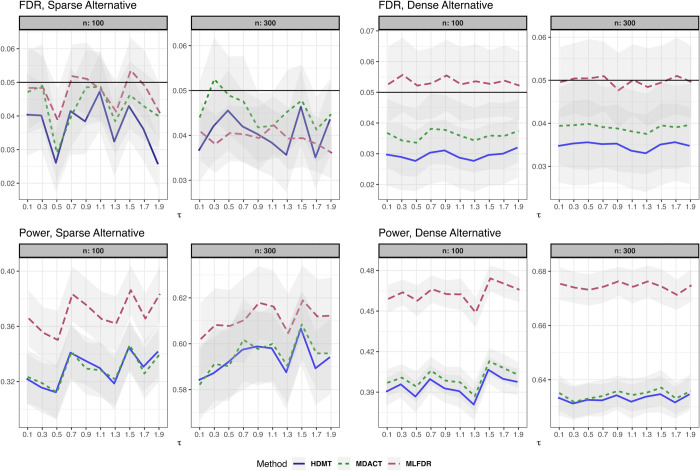
FDR and power comparison for the linear model (Setting 1). Results are displayed for both sparse and dense alternatives. Gray ribbons indicate error margins.

**Fig 3:**
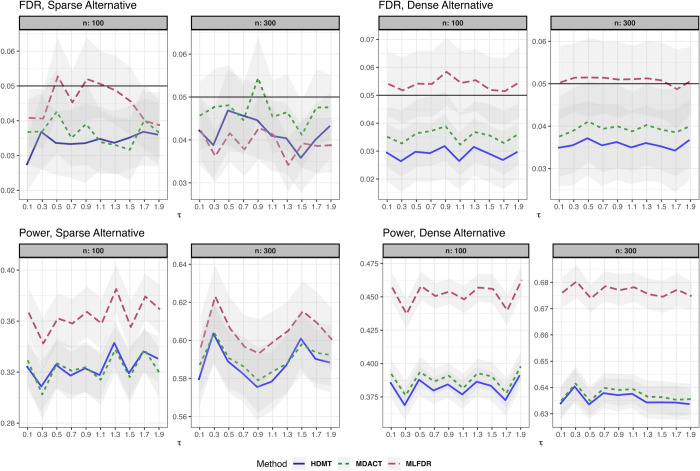
FDR and power comparison for the linear model with measured confounders (Setting 2). Results are displayed for both sparse and dense alternatives. Gray ribbons indicate error margins.

**Fig 4:**
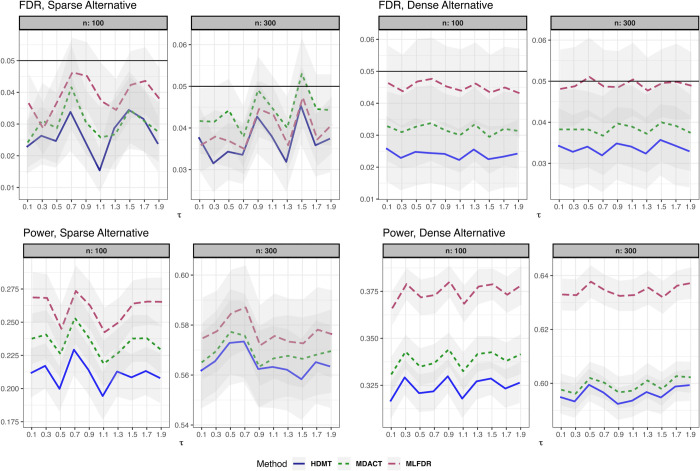
FDR and power comparison for the linear model with binary outcomes (Setting 3). Results are displayed for both sparse and dense alternatives. Gray ribbons indicate error margins.

1. **Linear Model.** The exposure is univariate with X∼Ber(0.1).
  $$M_{i}=X\alpha_{i}+e_{i},\;Y_{i}=M_{i}\beta_{i}+X\gamma_{i}+\epsilon_{i},$$ (3)
  where $\gamma_{i}∼N(1,0.5)$, and error terms $e_{i},\epsilon_{i}\overset{i.i.d.}{∼}N(0,1)$. The results for this setting are summarized in Fig 2.
2. **Linear Model with measured Confounder.** The exposure is univariate with X∼Ber(0.1). We introduce a confounder Z∼N(0,1):
  $$M_{i}=X\alpha_{i}+\theta_{i}Z+e_{i},\;Y_{i}=M_{i}\beta_{i}+X\gamma_{i}+\delta_{i}Z+\epsilon_{i},$$ (4)
  where the confounder effects are drawn independently from $\theta_{i},\delta_{i}∼U(0,0.5)$. The results are presented in Fig 3.
3. **Binary Outcome.** The exposure is univariate with X∼Ber(0.1). The outcome *Y*_*i*_ is binary:
  $$M_{i}=X\alpha_{i}+e_{i},\;\text{logit}\{\mathbb{P}(Y_{i}=1)\}=M_{i}\beta_{i}+X\gamma_{i}.$$ (5)
  The results are displayed in Fig 4.

Across all settings, the three methods demonstrated satisfactory FDR control. However, MLFDR consistently exhibited the highest power. Specifically, MLFDR achieved an average power improvement of 10.83% over MDACT and 12.23% over HDMT under dense alternatives. Under sparse alternatives, MLFDR maintained its advantage with an average improvement of 7.47% over MDACT and 8.51% over HDMT.

### 2.3 Extensions

#### 2.3.1 Latent factors.

Unmeasured latent factors may be addressed by surrogate variable analysis [10]. Briefly, surrogate variable analysis considers the following model:

$$M_{i}=\mu_{i}+\alpha_{i}X+\phi_{i}Z+\sum_{l=1}^{L}\gamma_{l}^{\left(i\right)}g_{l}+e_{i},$$ (6)

where *X*, *Z* are measured covariates, and $g_{1},g_{2},\ldots g_{L}$ are unmeasured latent factors. Surrogate variable analysis produces a set of *K* mutually orthogonal vectors ${\hat{u}}_{1},{\hat{u}}_{2},\ldots ,{\hat{u}}_{K}$ (where K≤L), which span the same linear space as the latent factors. Thus, the original equation may be re-written as:


$$M_{i}=\mu_{i}+\alpha_{i}X+\phi_{i}Z+\sum_{k=1}^{K}\lambda_{k}^{\left(i\right)}{\hat{u}}_{k}+e_{i}.$$

These estimated factors, collected into a matrix ${\hat{𝐔}}_{M}$, account for unmeasured confounding in the exposure-mediator relationship.

For the mediator-outcome relationship, the latent factors may be modeled as follows:

$$Y_{i}=\nu_{i}+\beta_{i}M_{i}+\gamma_{i}X+\delta_{i}Z+\sum_{l=1}^{L}\eta_{l}^{\left(i\right)}g_{l}+\epsilon_{i}.$$ (7)

In this setting, the latent terms may account for: 1) unmeasured confounding; 2) measured confounders with unknown relationships to the outcome (e.g., global batch effects); and 3) pleiotropy, where *Y*_*i*_ is influenced by mediators other than *M*_*i*_.

Direct application of SVA to model (7) would require estimating surrogate variables for each mediator-outcome pair iteratively, leading to a computational bottleneck. To address this, we propose a global factor adjustment. We estimate a second set of surrogate variables, ${\hat{𝐔}}_{Y}$, based on the outcome null model (excluding mediators):

$$Y_{i}=\nu_{i}+\gamma_{i}X+\delta_{i}Z+\sum_{l=1}^{L}\eta_{l}^{\left(i\right)}g_{l}+\epsilon_{i}.$$ (8)

We then use the combined set of latent factors ${\hat{𝐔}}_{M}$ (derived from mediators) and ${\hat{𝐔}}_{Y}$ (derived from outcome residuals) to model the mediator-outcome relationship. The validity of using ${\hat{𝐔}}_{Y}$ from (8) relies on the assumption that any single mediator *M*_*i*_ contributes a relatively small amount of variance to the global outcome matrix **Y**.

The full procedure is summarized in Algorithm 1. We implemented surrogate variable analysis via the Bioconductor package sva [9].

**Algorithm 1 Two-step global factor adjustment for high-dimensional mediation**

**Require:** Outcome matrix $𝐘\in {\mathbb{R}}^{n\times m}$, Mediator matrix $𝐌\in {\mathbb{R}}^{n\times m}$, Exposure vector $𝐗\in {\mathbb{R}}^{n\times 1}$, Covariates $𝐙\in {\mathbb{R}}^{n\times q}$.

**Ensure:** Adjusted estimates for ${\hat{\alpha }}_{i}$ and ${\hat{\beta }}_{i}$ for i=1,…,m.

  **Step 1: Estimate Latent Factors for Mediators (${\hat{𝐔}}_{M}$)**

1: Regress **M** on **X** and **Z** to obtain the residual matrix ${\hat{𝐑}}_{M}$:


$${\hat{𝐑}}_{M}=𝐌-\left(𝐗\hat{𝐀}+𝐙\hat{\Phi }\right)$$

2: Perform SVA on ${\hat{𝐑}}_{M}$ to extract the top *k*_*M*_ principal components:


$${\hat{𝐔}}_{M}\leftarrow \text{SVA}({\hat{𝐑}}_{M},k_{M})$$

  **Step 2: Estimate Latent Factors for Outcomes (${\hat{𝐔}}_{Y}$)**

3: Regress **Y** on **X** and **Z** (excluding **M**) to obtain the residual matrix ${\hat{𝐑}}_{Y}$:


$${\hat{𝐑}}_{Y}=𝐘-\left(𝐗\hat{\Gamma }+𝐙\hat{\Delta }\right)$$

4: Perform SVA on ${\hat{𝐑}}_{Y}$ to extract the top *k*_*Y*_ principal components:


$${\hat{𝐔}}_{Y}\leftarrow \text{SVA}({\hat{𝐑}}_{Y},k_{Y})$$

  **Step 3: Mediation Analysis with Global Adjustment**

5: **for** i=1,…,m **do**

6:   *Mediator Model:* Regress *M*_*i*_ on **X**, **Z**, and ${\hat{𝐔}}_{M}$:


$$M_{i}=𝐗\alpha_{i}+𝐙\phi_{i}+{\hat{𝐔}}_{M}\lambda_{M,i}+e_{i}$$

7:   *Outcome Model:* Regress *Y*_*i*_ on *M*_*i*_, **X**, **Z**, ${\hat{𝐔}}_{M}$, and ${\hat{𝐔}}_{Y}$:


$$Y_{i}=M_{i}\beta_{i}+𝐗\gamma_{i}+𝐙\delta_{i}+{\hat{𝐔}}_{M}\lambda_{Y1,i}+{\hat{𝐔}}_{Y}\lambda_{Y2,i}+\epsilon_{i}$$

8:   Extract coefficients ${\hat{\alpha }}_{i},{\hat{\beta }}_{i}$ and their standard errors for MLFDR.

9: **end for**

We apply this method to two data generating scenarios to demonstrate its performance.

**Fig 5:**
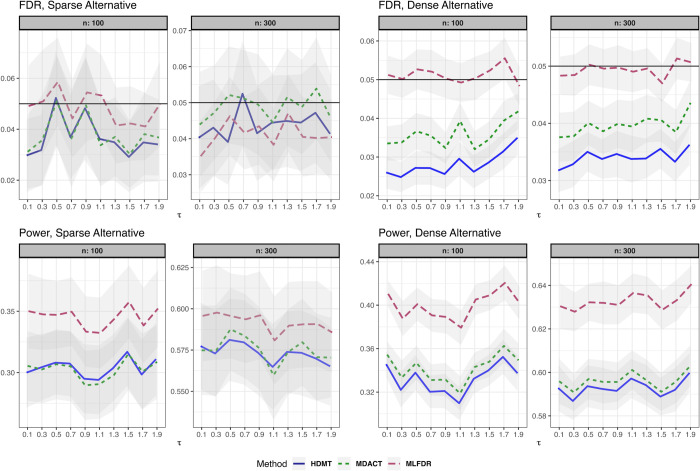
FDR and power comparison for the linear model with unknown mediator-exposure interactions. Results are displayed for both sparse and dense alternatives. Gray ribbons indicate error margins.

**Unknown mediator-exposure interactions.**

$$M_{i}=\alpha_{i}X+\delta_{i}Z+e_{i},\;Y_{i}=M_{i}\beta_{i}+X\gamma_{i}+\zeta_{i}Z+X\sum_{j\in S}\theta_{j}M_{j}+\epsilon_{i},$$ (9)

where *S* is a randomly selected subset of indices {1,…,m} with |S|=20, treated as unknown during model fitting. The results are shown in Fig 5.

**Fig 6:**
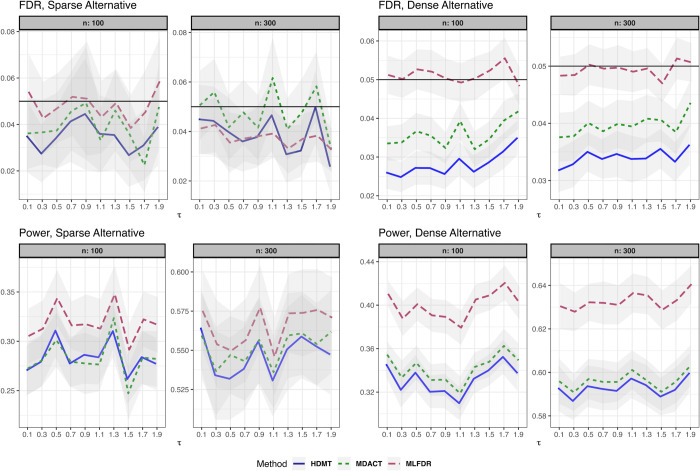
FDR and power comparison for the linear model with unmeasured confounders. Results are displayed for both sparse and dense alternatives. Gray ribbons indicate error margins.

**Unmeasured confounding and pleiotropy.**

$$M_{i}=X\alpha_{i}+\theta_{i}Z+0.4Z_{1}+0.5Z_{2}+e_{i},\;Y_{i}=M_{i}\beta_{i}+X\gamma_{i}+\delta_{i}Z-0.5Z_{1}+\sum_{j\in S}M_{j}\kappa_{j}+\epsilon_{i},$$ (10)

where *Z*_1_ and *Z*_2_ represent unmeasured confounders not included in the model fitting. *S* is a randomly selected subset of indices {1,…,m} with |S|=20. The term $\sum_{j\in S}M_{j}\kappa_{j}$ represents dense pleiotropy (the effect of other mediators on *Y*_*i*_), which acts as an additional source of unmeasured variation. The results are shown in Fig 6.

#### 2.3.2 Composite alternatives.

In this setting, the coefficients (α,β) follow a Gaussian mixture distribution. The posterior distribution of the estimated coefficients is given by:


$$\sqrt{n}{\hat{\alpha }}_{i}∼p_{0}N(0,\sigma_{i1}^{2})+p_{1}N(\mu_{1},\sigma_{i1}^{2}+\kappa_{1})+p_{2}N(\mu_{2},\sigma_{i1}^{2}+\kappa_{2}),\;\sqrt{n}{\hat{\beta }}_{i}∼q_{0}N(0,\sigma_{i2}^{2})+q_{1}N(\theta_{1},\sigma_{i2}^{2}+\psi_{1})+q_{2}N(\theta_{2},\sigma_{i2}^{2}+\psi_{2}).$$

Under this framework, the null hypothesis corresponds to a mixture of 5 bivariate Gaussian distributions, while the alternative hypothesis comprises a mixture of 4 bivariate Gaussian distributions. The parameters are estimated using a two-step EM algorithm, the details of which are provided in Section C of S1 Text.

In our simulations, we set the mixture weights to $\left(p_{0},p_{1},p_{2})=(0.54,0.18,0.28\right)$ and $\left(q_{0},q_{1},q_{2})=(0.6,0.05,0.35\right)$. The variance parameters were fixed at $\kappa_{1}=1,\kappa_{2}=2,\psi_{1}=1.5,\psi_{2}=2$. The mean parameters were defined as functions of the mediation signal strength *τ*: $\mu_{1}=0.05\tau$, $\mu_{2}=-0.5\tau$, $\theta_{1}=0.9\tau$, and $\theta_{2}=-0.01\tau$, where *τ* ranges from 0.1 to 1.9 in increments of 0.2.

**Fig 7:**
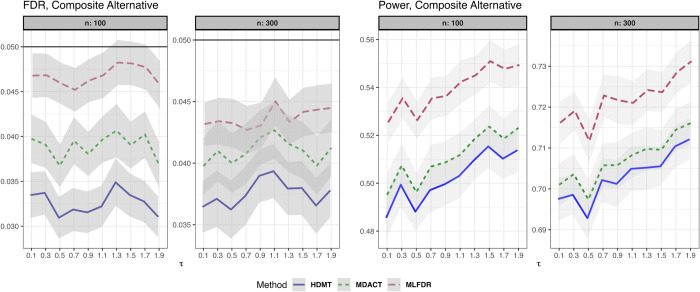
FDR and power comparison for composite alternatives. Results are displayed for varying degrees of mediation (*τ*). Gray ribbons indicate error margins.

Fig 7 presents the results for n∈{100,300} with *m* = 1000. All three methods maintained satisfactory FDR control, with MLFDR demonstrating the highest power.

All methods appear to have satisfactory FDR control. MLFDR is uniformly more powerful than HDMT and MDACT in all cases, with better margin of improvement in dense alternatives.

## 3 Real data analysis

### 3.1 TCGA prostate cancer data

We apply MLFDR to the Prostate Cancer dataset from The Cancer Genome Atlas (TCGA), previously analyzed by [3]. The study involves mediation analysis for 147 prostate cancer risk SNPs, integrated with DNA methylation and gene expression data from 495 samples. For each risk SNP, we identified CpG methylation probes within a 500 kb window and recorded the gene expression levels for the corresponding probes. This resulted in m=69,602 SNP-CpG-Gene triplets for mediation testing.

In the first stage, we regressed CpG methylation on the SNPs, adjusting for the top 3 principal components (PCs) of genotypes, the top 15 PCs of CpG methylation, age at diagnosis, and pathological stage. From this, we obtained the slope estimates, variances, and p-values for the SNPs. In the second stage, gene expression was regressed on CpG methylation, conditional on the same set of covariates.

The estimated null proportion components $\left(\pi_{00},\pi_{10},\pi_{01}\right)$ were (0.51,0.033,0.41) for HDMT, compared to (0.39,0.004,0.59) obtained via the EM algorithm in MLFDR.

**Fig 8:**
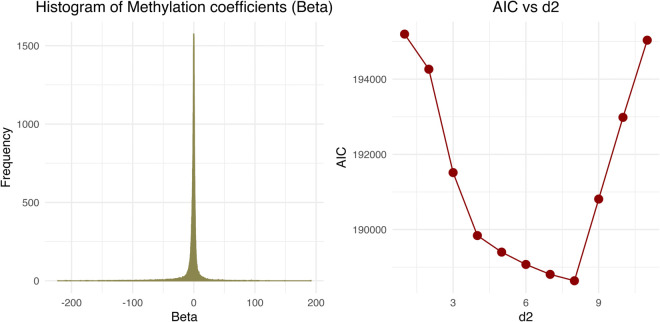
AIC for methylation coefficients when a *d*_2_ + 1 component Gaussian mixture model was fit.

Due to the wide spread of the methylation coefficients (*β*), we fitted a composite alternative Gaussian mixture model. The number of components, *d*_2_ = 8, was selected based on the Akaike Information Criterion (AIC) (Fig 8). Conversely, the SNP coefficients (*α*) exhibited a narrower range (–0.2 to 0.4) and were adequately modeled using a *d*_1_ = 2 component Gaussian mixture.

**Fig 9:**
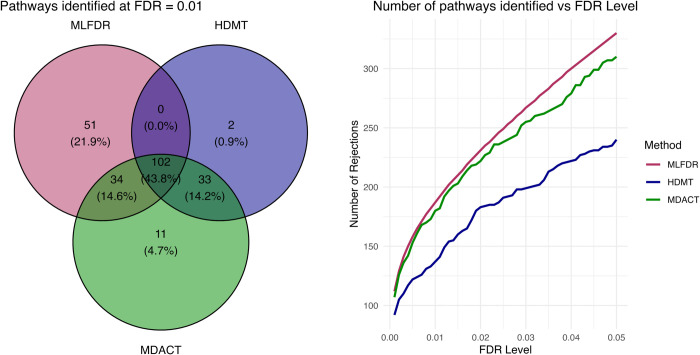
SNP-CpG-Gene triplets identified by MDACT, MLFDR, and HDMT out of 69,602 tests. The Venn diagram (left) corresponds to an FDR cutoff of 0.01.

At an FDR threshold of 0.01, HDMT identified 137 triplets, MLFDR identified 187, and MDACT identified 180. Fig 9 displays a Venn diagram of the overlapping discoveries, along with the number of rejections across FDR cutoffs ranging from 0.001 to 0.05. MLFDR consistently detected more pathways than the competing methods.

Table 1 lists 10 additional pathways identified by MLFDR (but missed by HDMT or MDACT), ranked by local FDR. Notably, six of these triplets involve rs12653946, a known prostate cancer risk variant that influences the expression of *IRX4*, a tumor suppressor gene in the prostate [6]. Another pathway involves rs7767188, a risk SNP associated with prostate cancer through the expression of *TRIM26* [29].

**Table 1:** Top 10 additional pathways detected by MLFDR in TCGA Prostate Cancer Data (ranked by $p_{\max}$). These pathways were not detected by HDMT or MDACT.

SNP	CpG Probe	Annotated Gene	Target Gene	$p_{\max}$	Local FDR
rs7767188	cg02749752	TRIM26	TRIM26	0.0017	0.0034
rs12653946	cg12830271	–	IRX4	0.0019	0.0058
rs3096702	cg19609334	TNXB	TNXB	0.0019	0.0060
rs3129859	cg03520342	HLA-DMA	HLA-DMA	0.0019	0.0125
rs12653946	cg00085370	–	IRX4	0.0022	0.0067
rs12653946	cg07144328	–	IRX4	0.0022	0.0063
rs12653946	cg07278634	–	IRX4	0.0028	0.0077
rs12653946	cg06446548	–	NDUFS6	0.0028	0.0288
rs12653946	cg03225093	–	IRX4	0.0029	0.0083
rs5945619	cg10581449	NUDT11	NUDT11	0.0030	0.0077

These empirical results align with our simulation studies: HDMT yielded the fewest discoveries, followed by MDACT, with MLFDR providing the highest detection rate. We note that the improvement of MLFDR over MDACT is modest in this application. This is likely attributable to the symmetric distribution of *α* and *β*; as noted by [22], local FDR-based tests offer limited power gains over p-value-based methods when the alternative distribution is symmetric around the null.

### 3.2 TCGA lung squamous cell carcinoma

We further extend our analysis to the TCGA Lung Squamous Cell Carcinoma (LUSC) dataset to investigate the mediating role of CpG methylation in the relationship between smoking history and gene expression. The data were acquired using the R package UCSCXenaTools [28]. We restricted the analysis to primary tumor samples, resulting in a sample size of *n* = 379 after preprocessing.

Smoking history was quantified by pack-years. Using the publicly available probe map data from the TCGA website, each CpG probe was mapped to the expression profiles of potentially multiple genes; each unique CpG-gene pair was treated as a distinct candidate mediation pathway. The analysis proceeded in two stages. In the first stage, CpG methylation beta values were regressed on smoking history (pack-years). In the second stage, a multiple linear regression was fitted for each gene, including all CpG probes mapped to that gene as predictors. The coefficients and p-values for each specific CpG-gene pair were then extracted to form the mediation hypotheses. All models were adjusted for potential confounders, including sex and the age at initial diagnosis. In total, 319,761 CpG-gene pathways were evaluated.

**Fig 10:**
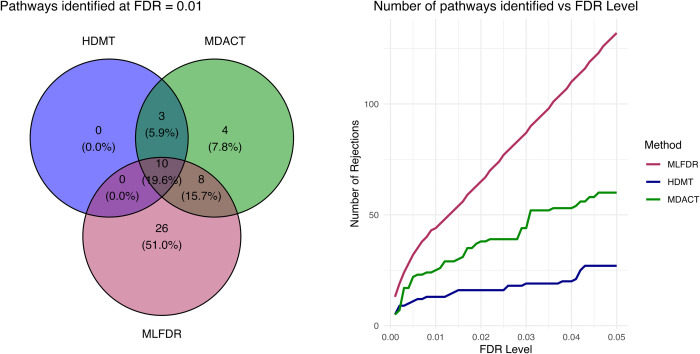
Smoking-CpG-Gene pathways detected by MDACT, MLFDR, and HDMT out of 319,761 tests. The Venn diagram (left) displays overlaps at an FDR cutoff of 0.01, while the plot (right) shows detection counts across varying FDR thresholds.

At an FDR threshold of 0.01, HDMT identified 13 pathways, MDACT identified 25 pathways, and MLFDR identified 44 pathways (Fig 10). The results highlight the increased power of MLFDR in detecting subtle mediation signals. Table 2 details the top 10 additional pathways detected by MLFDR (ranked by local FDR) that were not identified by the competing methods. Several of these findings are supported by existing literature. For instance, [27] discuss the relevance of *WDR66* in lung cancer progression, while [2] highlights the role of *LY6K* as a potential therapeutic target in lung squamous cell carcinoma. [31] links *TCIRG1* to metastatic potential of hepatocellular carcinoma.

**Table 2:** Top 10 additional pathways detected by MLFDR in the TCGA LUSC dataset (ranked by $p_{\max}$). These pathways were not detected by HDMT or MDACT.

Rank	Gene	CpG Probe	$p_{\max}$	Local FDR
1	HOXB6	cg20591728	$6.6\times 10^{-6}$	0.0039
2	MYLIP	cg04641165	$1.7\times 10^{-5}$	0.0040
3	B3GALT2	cg16712103	$1.8\times 10^{-5}$	0.0100
4	ZNF287	cg16964464	$2.2\times 10^{-5}$	0.0085
5	TCIRG1	cg20484322	$3.6\times 10^{-5}$	0.0131
6	WDR66	cg03560652	$3.8\times 10^{-5}$	0.0145
7	ENO3	cg07333510	$4.5\times 10^{-5}$	0.0139
8	ADORA2B	cg21501163	$5.8\times 10^{-5}$	0.0197
9	GNA14	cg06617692	$6.4\times 10^{-5}$	0.0231
10	LY6K	cg16809304	$8.1\times 10^{-5}$	0.0148

**Fig 11:**
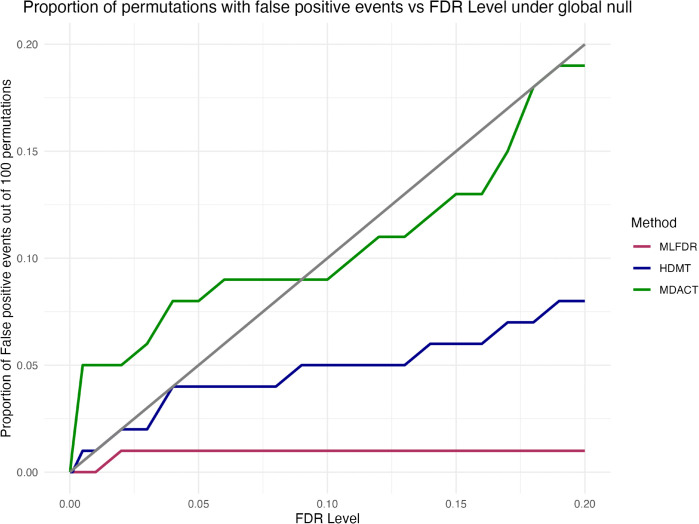
Smoking-CpG-gene pathways detected by all methods under the global null out of m=1,000 tests. The proportion of cases reporting positive detection counts is reported across varying FDR thresholds. The gray solid line indicates target FDR levels.

To evaluate the FDR of the three methods, we selected a subset of the data with m=1,000 tests, and permuted the samples to create a “global null” scenario. For each permutation, the smoking-Cpg and CpG-gene expression model is fit, and the three methods are implemented. At a given permutation, if any method has non-zero rejections, the False Positive rate for that permutation is recorded as 1 for that method. This procedure is repeated over 100 permutations, and the number of rejections is recorded for FDR levels varying from 0.0001 to 0.2. Fig 11 presents the proportion of cases in which each method came up with significant rejections under the global null. HDMT and MLFDR appear to have satisfactory control of the FDR, while MDACT appears to have some FDR inflation at low FDR levels.

## 4 Discussion

We have presented a flexible and powerful screening algorithm for detecting causal pathways in high-dimensional mediation analysis. MLFDR is capable of dealing with a wide array of outcome variables, confounders, and mediation exposure interactions. It can work in tandem with surrogate variable analysis to address complex dependence structures like pleiotropy and unmeasured confounding. Through simulations and theoretical analysis, we have shown that the proposed method is a viable alternative to p-value-based screening methods, which may not produce optimal results depending on the structure of the alternative hypothesis. We have also shown theoretical guarantees of FDR control for MLFDR. Overall, we hope that MLFDR will be used as an alternative to p-value based methods for mediator screening in the future.

## 5 Methods

### 5.1 The mediation model

Consider a univariate exposure *X*, a set of mediators $\{M_{i}{\}}_{i=1}^{m}$, and *m* outcomes $\{Y_{i}{\}}_{i=1}^{m}$. We assume the following structural equation model:

$$M_{i}=X\alpha_{i}+e_{i},\;Y_{i}=M_{i}\beta_{i}+X\gamma_{i}+\epsilon_{i},$$ (11)

where the error terms satisfy $\left(e_{i},\epsilon_{i})⟂(X,M_{i}\right)$ and are distributed as:


$$(e_{i},\epsilon_{i})∼N\left((\begin{array}{c} 0\\ 0 \end{array}),(\begin{array}{cc} \sigma_{i,a}^{2} & 0\\ 0 & \sigma_{i,b}^{2} \end{array})\right).$$

In settings with high-dimensional exposures, such as Single Nucleotide Polymorphisms (SNPs), we define the hypothesis based on the Exposure-Mediator-Outcome triplet $\{X_{i},M_{i},Y_{i}{\}}_{i=1}^{m}$. In such cases, the common exposure *X* in Eq (11) is replaced by the specific exposure *X*_*i*_.

To describe the methodology, we focus on the formulation in Model (11). Given *n* independent samples $\{(Y^{\;j},X^{\;j},M^{\;j}){\}}_{j=1}^{n}$, our goal is to test the joint significance of $\alpha_{i}$ and $\beta_{i}$ for 1≤i≤m. Let ${𝐘}_{i}=(Y_{i}^{1},\ldots ,Y_{i}^{\;n}{)}^{\prime }$, $𝐗=(X^{1},\ldots ,X^{\;n}{)}^{\prime }$, ${𝐌}_{i}=(M_{i}^{1},\ldots ,M_{i}^{\;n}{)}^{\prime }$, and define the projection matrix $𝐏=𝐈-𝐗({𝐗}^{\prime }𝐗{)}^{-1}{𝐗}^{\prime }$. The ordinary least squares (OLS) estimators are given by:

$${\hat{\alpha }}_{i}=({𝐗}^{\prime }𝐗{)}^{-1}{𝐗}^{\prime }{𝐌}_{i},\;{\hat{\beta }}_{i}=({𝐌}_{i}^{\prime }𝐏{𝐌}_{i}{)}^{-1}{𝐌}_{i}^{\prime }𝐏{𝐘}_{i}.$$ (12)

Conditional on **X** and ${𝐌}_{i}$, the estimators follow the distribution:


$$\sqrt{n}(\begin{array}{c} {\hat{\alpha }}_{i}-\alpha_{i}\\ {\hat{\beta }}_{i}-\beta_{i} \end{array})=\sqrt{n}(\begin{array}{c} ({𝐗}^{\prime }𝐗{)}^{-1}{𝐗}^{\prime }{𝐞}_{i}\\ ({𝐌}_{i}^{\prime }𝐏{𝐌}_{i}{)}^{-1}{𝐌}_{i}^{\prime }𝐏\epsilon_{i} \end{array})\;\overset{d}{=}N\left((\begin{array}{c} 0\\ 0 \end{array}),(\begin{array}{cc} \sigma_{i1}^{2} & 0\\ 0 & \sigma_{i2}^{2} \end{array})\right),$$

where $\sigma_{i1}^{2}=n\sigma_{i,a}^{2}({𝐗}^{\prime }𝐗{)}^{-1}$ and $\sigma_{i2}^{2}=n\sigma_{i,b}^{2}({𝐌}_{i}^{\prime }𝐏{𝐌}_{i}{)}^{-1}$. Note that ${\hat{\alpha }}_{i}$ and ${\hat{\beta }}_{i}$ are independent, as $𝔼[({𝐗}^{\prime }𝐗{)}^{-1}{𝐗}^{\prime }{𝐞}_{i}\epsilon_{i}^{\prime }𝐏{𝐌}_{i}({𝐌}_{i}^{\prime }𝐏{𝐌}_{i}{)}^{-1}]=0$. We test the composite null hypothesis against the alternative:

$$H_{0,i}:\alpha_{i}\beta_{i}=0\;\text{versus}\;H_{11,i}:\alpha_{i}\beta_{i}\neq 0,\;i=1,2,\ldots ,m.$$ (13)

Denote by $\xi_{i}=(\xi_{i1},\xi_{i2})$ the latent vector indicating the underlying truth of the *i*-th hypothesis, where $\xi_{i1}=1\{\alpha_{i}\neq 0\}$ and $\xi_{i2}=1\{\beta_{i}\neq 0\}$. The vector $\xi_{i}$ takes values in the set {(0,0),(0,1),(1,0),(1,1)}. Let $m_{jk}=\sum_{i=1}^{m}1\{\xi_{i}=(j,k)\}$ represent the count of hypotheses in each state for 0≤j,k≤1.

We assume a prior distribution $P(\xi_{i}=(j,k))=\pi_{jk}$, where $\pi_{jk}\geq 0$ and $\sum_{j,k}\pi_{jk}=1$. Conditional on the latent states, the non-zero effect sizes are modeled as Gaussian:


$$\sqrt{n}\alpha_{i}|(\xi_{i1}=1)∼N(\mu ,\psi )\;\text{and}\;\sqrt{n}\beta_{i}|(\xi_{i2}=1)∼N(\theta ,\kappa ).$$

When $\xi_{i1}=0$, $\alpha_{i}=0$ (and analogously for $\beta_{i}$). Assuming that $\alpha_{i}$ and $\beta_{i}$ are independent conditional on $\xi_{i}$, the marginal distribution of the coefficient estimates $\left({\hat{\alpha }}_{i},{\hat{\beta }}_{i}\right)$ given the latent states is:

$$\sqrt{n}(\begin{array}{c} {\hat{\alpha }}_{i}\\ {\hat{\beta }}_{i} \end{array})\;|\;\xi_{i}∼N\left((\begin{array}{c} \mu \xi_{i1}\\ \theta \xi_{i2} \end{array}),(\begin{array}{cc} \sigma_{i1}^{2}+\psi \xi_{i1} & 0\\ 0 & \sigma_{i2}^{2}+\kappa \xi_{i2} \end{array})\right),$$ (14)

where $\left(\sigma_{i1}^{2}/n,\sigma_{i2}^{2}/n\right)$ denote the variances of the estimates $\left({\hat{\alpha }}_{i},{\hat{\beta }}_{i}\right)$ conditional on $\left(\alpha_{i},\beta_{i}\right)$.

We employ an Expectation-Maximization (EM) algorithm to estimate the unknown parameters Θ={π,μ,θ,ψ,κ}; details are provided in Section B of the S1 Text. Although MLFDR is derived under the framework of Model (11), it is applicable to a broader range of settings, including binary outcomes, mediator-exposure interactions, and models with confounders. The extensive simulations presented earlier demonstrate the method’s robustness across these varied scenarios.

### 5.2 Extension: Composite alternative

In the last section we introduced the latent variable $\xi_{i}=(\xi_{i1},\xi_{i2})$ to characterize the underlying state of the hypothesis. We now extend this framework to a composite alternative setting, where the non-zero effects are drawn from mixture distributions. Specifically, we assume $\left(\alpha_{i},\beta_{i}\right)$ are generated as follows:


$$\sqrt{n}\alpha_{i}|\{\xi_{i1}=u\}∼N(\mu_{u},\kappa_{u}),\;u=0,1,\ldots ,d_{1},$$

$$\sqrt{n}\beta_{i}|\{\xi_{i2}=v\}∼N(\theta_{v},\psi_{v}),\;v=0,1,\ldots ,d_{2}.$$

Here, the index 0 denotes the null state, such that $\mu_{0}=\theta_{0}=\kappa_{0}=\psi_{0}=0$ (i.e., a degenerate distribution at zero). As before, $\alpha_{i}$ and $\beta_{i}$ are assumed independent conditional on the latent state $\xi_{i}$.

Marginalizing over the prior distribution, the joint distribution of the estimators $\left({\hat{\alpha }}_{i},{\hat{\beta }}_{i}\right)$ follows a Gaussian Mixture Model (GMM):

$$\sqrt{n}(\begin{array}{c} {\hat{\alpha }}_{i}\\ {\hat{\beta }}_{i} \end{array})∼\sum_{u=0}^{d_{1}}\sum_{v=0}^{d_{2}}\pi_{uv}N\left((\begin{array}{c} \mu_{u}\\ \theta_{v} \end{array}),(\begin{array}{cc} \sigma_{i1}^{2}+\kappa_{u} & 0\\ 0 & \sigma_{i2}^{2}+\psi_{v} \end{array})\right),$$ (15)

where $\pi_{uv}=P(\xi_{i1}=u,\xi_{i2}=v)$. In this context, the component null hypotheses map to specific index combinations: *H*_01,*i*_ corresponds to $\left\{\xi_{i1}=0,\xi_{i2}\in \{1,\ldots ,d_{2}\}\right\}$, while the alternative *H*_11,*i*_ corresponds to $\left\{\xi_{i1}\in \{1,\ldots ,d_{1}\},\xi_{i2}\in \{1,\ldots ,d_{2}\}\right\}$.

The corresponding marginal distributions are given by:

$$\sqrt{n}{\hat{\alpha }}_{i}∼\sum_{u=0}^{d_{1}}\pi_{u\cdot }N(\mu_{u},\sigma_{i1}^{2}+\kappa_{u}),\;\sqrt{n}{\hat{\beta }}_{i}∼\sum_{v=0}^{d_{2}}\pi_{\cdot v}N(\theta_{v},\sigma_{i2}^{2}+\psi_{v}),$$ (16)

where $\pi_{u\cdot }=\sum_{v}\pi_{uv}$ and $\pi_{\cdot v}=\sum_{u}\pi_{uv}$.

#### Two-step EM algorithm.

Directly fitting a (*d*_1_  +  1)(*d*_2_  +  1)-component bivariate GMM to estimate the joint probability matrix π is computationally intensive. To mitigate this burden, we propose a two-step Expectation-Maximization (EM) algorithm.

In the first step, we estimate the parameters $\{\mu_{u},\kappa_{u}{\}}_{u}$ and $\{\theta_{v},\psi_{v}{\}}_{v}$ using the univariate marginal distributions described in (Eq 16). While the marginal mixing proportions $\pi_{u\cdot }$ and $\pi_{\cdot v}$ are insufficient to recover the joint distribution $\pi_{uv}$ (without assuming independence), the moment estimates remain valid.

In the second step, we fix these mean and variance estimates and fit a constrained bivariate GMM to the joint data solely to estimate the joint mixing proportions $\pi_{uv}$. This approach significantly reduces the dimensionality of the optimization problem. Details of this algorithm are provided in Section C of S1 Text, and a supporting simulation is presented in Sect 2.3.

This two-step approach is also applicable to the standard case where $d_{1}=d_{2}=1$. In Section D of S1 Text, we compare the standard bivariate EM against this two-step variant. The results indicate that while the two-step method offers substantial computational speedups, it incurs a slight reduction in power. Therefore, we recommend using the standard bivariate EM for simple cases ($d_{1}=d_{2}=1$) and reserving the two-step EM for complex composite alternatives where computational efficiency is paramount.

### 5.3 Step-up procedure based on local FDR

[22] demonstrated that for a simple null hypothesis, the oracle local FDR-based rejection region outperforms p-value-based thresholding. Specifically, it achieves a lower marginal False Non-discovery Rate (mFNR) while controlling the marginal False Discovery Rate (mFDR) at the same level. This advantage is particularly pronounced when the alternative distribution is asymmetric about the null. Motivated by these findings, we implement a local FDR-based step-up procedure to identify significant mediation pathways.

We focus our discussion on the Gaussian Mixture Model described in (14). The extension to the more general mixture model in (15) is straightforward. Conditional on the latent variables, the density function of the transformed statistics $\sqrt{n}({\hat{\alpha }}_{i},{\hat{\beta }}_{i})$ under state *H*_*jk*,*i*_ is given by:

$$f_{jk}(\cdot ,\cdot ):=\phi (\cdot ,\cdot ;\mu \delta_{j},\theta \delta_{k},\sigma_{i1}^{2}+\kappa \delta_{j},\sigma_{i2}^{2}+\psi \delta_{k}),\;f:=\sum_{j=0}^{1}\sum_{k=0}^{1}\pi_{jk}f_{jk},$$ (17)

where $\delta_{0}=0$, $\delta_{1}=1$, and $\phi (\cdot ,\cdot ;\mu_{1},\mu_{2},\sigma_{1}^{2},\sigma_{2}^{2})$ denotes the bivariate normal density with mean vector $\left(\mu_{1},\mu_{2}\right)$, variances $\left(\sigma_{1}^{2},\sigma_{2}^{2}\right)$, and zero correlation.

For notational simplicity, let $a_{i}=\sqrt{n}{\hat{\alpha }}_{i}$ and $b_{i}=\sqrt{n}{\hat{\beta }}_{i}$. Under the composite null hypothesis, the joint local FDR is defined as:

$$\text{lfdr}(a_{i},b_{i})=\frac{\pi_{00}f_{00}(a_{i},b_{i})+\pi_{10}f_{10}(a_{i},b_{i})+\pi_{01}f_{01}(a_{i},b_{i})}{f(a_{i},b_{i})}.$$ (18)

This quantity can be estimated by substituting the parameter estimates obtained from the EM algorithm; we denote this estimate by $\hat{\text{lfdr}}$. As the local FDR represents the posterior probability of the null hypothesis, a lower value indicates stronger evidence against the null. Consequently, we define the rejection region as $\hat{\text{lfdr}}(a_{i},b_{i})\leq \delta$, where the threshold *δ* must be determined to control the error rate.

We assume the cumulative distribution function (CDF) of the joint local FDR is given by:

$$G(t)=\pi_{00}G_{00}(t)+\pi_{10}G_{10}(t)+\pi_{01}G_{01}(t)+\pi_{11}G_{11}(t),$$ (19)

where *G*_*jk*_(*t*) is the conditional CDF of $\text{lfdr}(a_{i},b_{i})$ under hypothesis *H*_*jk*_:

$$G(t)=\frac{1}{m}\sum_{i=1}^{m}\mathbb{P}(\text{lfdr}(a_{i},b_{i})\leq t),$$ (20)

$$G_{jk}(t)=\frac{1}{m}\sum_{i=1}^{m}\mathbb{P}(\text{lfdr}(a_{i},b_{i})\leq t|H_{jk}),\;j,k\in \{0,1\}.$$ (21)

#### Oracle procedure.

The oracle procedure assumes that all parameters $\pi =\{\pi_{00},\pi_{10},\pi_{01},\pi_{11}\}$ and densities *f*_*jk*_ are known. For a given threshold *δ*, we define the following counting processes:

$$V_{m}(\delta )=\sum_{i\in H_{00}\cup H_{10}\cup H_{01}}1\{\text{lfdr}(a_{i},b_{i})\leq \delta \},$$ (22)

$$R_{m}(\delta )=\sum_{i=1}^{m}1\{\text{lfdr}(a_{i},b_{i})\leq \delta \},$$ (23)

$$P_{m}(\delta )=\sum_{i\in H_{11}}1\{\text{lfdr}(a_{i},b_{i})>\delta \},$$ (24)

$$W_{m}(\delta )=\sum_{i=1}^{m}1\{\text{lfdr}(a_{i},b_{i})\leq \delta \}\cdot \text{lfdr}(a_{i},b_{i}),$$ (25)

where $V_{m}(\delta )$ represents the number of false rejections, $R_{m}(\delta )$ the total number of rejections, and $P_{m}(\delta )$ the number of missed discoveries (false negatives). We can express $V_{m}(\delta )$ as:


$$V_{m}(\delta )=\sum_{i=1}^{m}1\{\text{lfdr}(a_{i},b_{i})\leq \delta \}\cdot 1\{\xi_{i}\in \{(0,0),(1,0),(0,1)\}\}.$$

Taking the expectation, we obtain:

$$E[V_{m}(\delta )]=\sum_{i=1}^{m}E\left[1\{\text{lfdr}(a_{i},b_{i})\leq \delta \}\cdot 1\{\xi_{i}\in \{(0,0),(1,0),(0,1)\}\}\right]\;=m_{00}G_{00}(\delta )+m_{10}G_{10}(\delta )+m_{01}G_{01}(\delta ).$$ (26)

The mFDR at threshold *δ* is defined as:

$$\tilde{Q}(\delta )=\frac{E[V_{m}(\delta )]}{E[R_{m}(\delta )]}=\frac{\pi_{00}G_{00}(\delta )+\pi_{10}G_{10}(\delta )+\pi_{01}G_{01}(\delta )}{G(\delta )}.$$ (27)

This quantity can be empirically estimated by:

$$Q_{m}(\delta )=\frac{\sum_{i=1}^{m}1\{\text{lfdr}(a_{i},b_{i})\leq \delta \}\text{lfdr}(a_{i},b_{i})}{\sum_{i=1}^{m}1\{\text{lfdr}(a_{i},b_{i})\leq \delta \}}.$$ (28)

In Section A of the S1 Text, we prove that for a fixed *δ*, the numerator and denominator of $Q_{m}(\delta )$ are unbiased estimators of the numerator and denominator of $\tilde{Q}(\delta )$, respectively. The oracle rejection region for the composite null is defined as $1\{\text{lfdr}(a_{i},b_{i})\leq \delta_{m}\}$, where the threshold is selected as:

$$\delta_{m}=\sup\{t\in (0,1):Q_{m}(t)\leq \alpha \}.$$ (29)

#### Adaptive procedure.

In practical applications, the true parameters $\pi =\{\pi_{00},\pi_{10},\pi_{01},\pi_{11}\}$ and the component densities *f*_*jk*_ are unknown. Consequently, the true local FDR values in Equation (28) are inaccessible. To address this, we substitute the unknown quantities with their estimates obtained via the EM algorithm. By plugging in these estimates, we obtain the estimated local FDR, denoted as $\hat{\text{lfdr}}$, which yields an empirical estimate of $Q_{m}(\delta )$:

$${\hat{Q}}_{m}(\delta )=\frac{\sum_{i=1}^{m}1\{\hat{\text{lfdr}}(a_{i},b_{i})\leq \delta \}\hat{\text{lfdr}}(a_{i},b_{i})}{\sum_{i=1}^{m}1\{\hat{\text{lfdr}}(a_{i},b_{i})\leq \delta \}}.$$ (30)

Accordingly, the data-adaptive threshold ${\hat{\delta }}_{m}$ is defined as:


$${\hat{\delta }}_{m}=\sup\{t\in (0,1):{\hat{Q}}_{m}(t)\leq \alpha \}.$$

The resulting procedure, which rejects the *i*-th hypothesis if $\hat{\text{lfdr}}(a_{i},b_{i})\leq {\hat{\delta }}_{m}$, is operationally equivalent to the step-up algorithm detailed in Algorithm 2.

**Algorithm 2 A data-adaptive procedure for finding the cutoffs in MLFDR.**

**Require:** EM estimates for the parameters Θ={π,μ,θ,ψ,κ}

1: **for** i=1,…,m **do**

2:   Compute ${\hat{\text{lfdr}}}_{i}=\hat{\text{lfdr}}(\sqrt{n}{\hat{\alpha }}_{i},\sqrt{n}{\hat{\beta }}_{i})$

3: **end for**

4: Sort the estimated local FDR values in ascending order to obtain the set of order statistics $\Psi =\{{\hat{\text{lfdr}}}_{\left(1\right)},{\hat{\text{lfdr}}}_{\left(2\right)},\ldots ,{\hat{\text{lfdr}}}_{\left(m\right)}\}$, where ties are broken at random.

5: For each k∈{1,…,m}, compute the average estimated local FDR for the top *k* statistics:


$${\hat{Q}}_{m}({\hat{\text{lfdr}}}_{\left(k\right)})=\frac{1}{k}\sum_{i=1}^{k}{\hat{\text{lfdr}}}_{\left(i\right)}.$$

6: Determine the cutoff index $k=\max\left\{j:\frac{1}{j}\sum_{i=1}^{\;j}{\hat{\text{lfdr}}}_{\left(i\right)}\leq \alpha \right\}$

7: Reject the null hypotheses corresponding to the smallest *k* local FDR values, denoted as $H_{\left(1\right)},H_{\left(2\right)},\ldots ,H_{\left(k\right)}$

## Supporting information

S1 Fig Comparison of empirical FDR and power across different methods for MLFDR and two-step MLFDR. (TIFF)

S1 Text Supplementary file detailing the methods and the theory. (PDF)
