# Supplementary material for: Powerful large scale inference in high dimensional mediation analysis
Source: PLoS Comput Biol. 2026 Jan 14;22(1):e1013880. doi: 10.1371/journal.pcbi.1013880 (PMC12829953; doi:10.1371/journal.pcbi.1013880)
Supplement: S1 Text — (PDF) [file pcbi.1013880.s002.pdf]

Supplement to "Powerful Large Scale Inference in High Dimensional Mediation Analysis"

Asmita Roy[1], Xianyang Zhang[2,*]

1 Department of Biostatistics/Bloomberg School of Public Health, Johns Hopkins University, Baltimore, Maryland, USA
2 Department of Statistics, Texas A&M University, College Station, Texas, USA

A Discussions on the step-up procedure

Proposition 1. *Let G_{00}, G_{10}, G_{01} be as defined in the main paper. We have*

$$E\left[\frac{1}{m}\sum_{i=1}^{m} I\{lfdr(\sqrt{n}\hat{\alpha}_i, \sqrt{n}\hat{\beta}_i) \leq \delta\} lfdr(\sqrt{n}\hat{\alpha}_i, \sqrt{n}\hat{\beta}_i)\right]$$
$$= \pi_{00}G_{00}(\delta) + \pi_{10}G_{10}(\delta) + \pi_{01}G_{01}(\delta).$$

Proof. Define $S_i^n(\delta) = \{(\hat{\alpha}_i, \hat{\beta}_i) : \mathrm{lfdr}(\sqrt{n}\hat{\alpha}_i, \sqrt{n}\hat{\beta}_i) \leq \delta\}$. Note that

$$\begin{aligned}
&E\left[\sum_{i=1}^{m} I\{\mathrm{lfdr}(\sqrt{n}\hat{\alpha}_i, \sqrt{n}\hat{\beta}_i) \leq \delta\}\mathrm{lfdr}(\sqrt{n}\hat{\alpha}_i, \sqrt{n}\hat{\beta}_i)\right] \\
&= \sum_{i=1}^{m} E\left[I\{\mathrm{lfdr}(\sqrt{n}\hat{\alpha}_i, \sqrt{n}\hat{\beta}_i) \leq \delta\}\mathrm{lfdr}(\sqrt{n}\hat{\alpha}_i, \sqrt{n}\hat{\beta}_i)\right] \\
&= \sum_{i=1}^{m} \int_{S_i^n(\delta)} \mathrm{lfdr}(a_i, b_i) f(a_i, b_i) da_i b_i \\
&= \sum_{i=1}^{m} \int_{S_i^n(\delta)} \pi_{00} f_{00}(a_i, b_i) da_i b_i + \sum_{i=1}^{m} \int_{S_i^n(\delta)} \pi_{10} f_{10}(a_i, b_i) da_i b_i \\
&+ \sum_{i=1}^{m} \int_{S_i^n(\delta)} \pi_{01} f_{01}(a_i, b_i) da_i b_i \\
&= \pi_{00} \sum_{i=1}^{m} \mathbb{P}(\mathrm{lfdr}(\sqrt{n}\hat{\alpha}_i, \sqrt{n}\hat{\beta}_i) \leq t | X, \{M_i\}_i, H_{00}) \\
&+ \pi_{10} \sum_{i=1}^{m} \mathbb{P}(\mathrm{lfdr}(\sqrt{n}\hat{\alpha}_i, \sqrt{n}\hat{\beta}_i) \leq t | X, \{M_i\}_i, H_{10}) \\
&+ \pi_{01} \sum_{i=1}^{m} \mathbb{P}(\mathrm{lfdr}(\sqrt{n}\hat{\alpha}_i, \sqrt{n}\hat{\beta}_i) \leq t | X, \{M_i\}_i, H_{01}) \\
&= m_{00}G_{00}(\delta) + m_{10}G_{10}(\delta) + m_{01}G_{01}(\delta).
\end{aligned}$$

□

From Proposition 1, and noting that

$$\mathbb{E}\left[\frac{1}{m} I\{\text{lfdr}(\sqrt{n}\hat{\alpha}_i, \sqrt{n}\hat{\beta}_i) \le \delta\}\right] = G(\delta),$$

it appears reasonable to estimate

$$\widetilde{Q}(\delta) = \frac{\pi_{00}G_{00}(\delta) + \pi_{10}G_{10}(\delta) + \pi_{01}G_{01}(\delta)}{G(\delta)},$$

by

$$Q_m(\delta) = \frac{\sum_{i=1}^m I\{\text{lfdr}(a_i, b_i) \le \delta\}\text{lfdr}(a_i, b_i)}{\sum_{i=1}^m I\{\text{lfdr}(a_i, b_i) \le \delta\}}.$$

B EM Algorithm Details

Let $\Gamma = (\pi_{00}, \pi_{10}, \pi_{01}, \pi_{11}, \mu, \theta, \kappa, \psi)$ denote the set of unknown parameters. We define the component densities corresponding to the four latent states as follows:

$$\begin{aligned}
f_{00}(\sqrt{n}\hat{\alpha}_i, \sqrt{n}\hat{\beta}_i) &:= \phi(\sqrt{n}\hat{\alpha}_i, \sqrt{n}\hat{\beta}_i; 0, 0, \sigma_{i1}^2, \sigma_{i2}^2), \\
f_{10}(\sqrt{n}\hat{\alpha}_i, \sqrt{n}\hat{\beta}_i) &:= \phi(\sqrt{n}\hat{\alpha}_i, \sqrt{n}\hat{\beta}_i; \mu, 0, \sigma_{i1}^2 + \kappa, \sigma_{i2}^2), \\
f_{01}(\sqrt{n}\hat{\alpha}_i, \sqrt{n}\hat{\beta}_i) &:= \phi(\sqrt{n}\hat{\alpha}_i, \sqrt{n}\hat{\beta}_i; 0, \theta, \sigma_{i1}^2, \sigma_{i2}^2 + \psi), \\
f_{11}(\sqrt{n}\hat{\alpha}_i, \sqrt{n}\hat{\beta}_i) &:= \phi(\sqrt{n}\hat{\alpha}_i, \sqrt{n}\hat{\beta}_i; \mu, \theta, \sigma_{i1}^2 + \kappa, \sigma_{i2}^2 + \psi),
\end{aligned} \tag{S2.1}$$

where $\phi(\cdot, \cdot; \mu_1, \mu_2, \sigma_1^2, \sigma_2^2)$ denotes the bivariate normal density with mean vector (μ_1, μ_2), variances (σ_1^2, σ_2^2), and zero correlation. The OLS variance estimates $(\sigma_{i1}^2, \sigma_{i2}^2)$ are treated as fixed inputs in the algorithm.

The complete data log-likelihood is given by:

$$\begin{aligned}
L(\Gamma) = &\sum_{i=1}^m \sum_{u,v\in\{0,1\}} \mathbb{1}(\boldsymbol{\xi}_i = (u,v)) \log f_{uv}(\sqrt{n}\hat{\alpha}_i, \sqrt{n}\hat{\beta}_i) \\
&+ \sum_{i=1}^m \sum_{u,v\in\{0,1\}} \mathbb{1}(\boldsymbol{\xi}_i = (u,v)) \log \pi_{uv}.
\end{aligned} \tag{S2.2}$$

E-Step

In the Expectation step, we calculate the conditional probability (posterior) of the latent variable $\boldsymbol{\xi}_i$ given the observed data $(\hat{\alpha}_i, \hat{\beta}_i)$ and the current parameter estimates $\Gamma^{(t)}$:

$$\begin{aligned}
Q_{i;u,v}^{(t)} &:= \mathbb{P}(\boldsymbol{\xi}_i = (u,v) \mid \hat{\alpha}_i, \hat{\beta}_i, \Gamma^{(t)}) \\
&= \frac{\pi_{uv}^{(t)} f_{uv}^{(t)}(\sqrt{n}\hat{\alpha}_i, \sqrt{n}\hat{\beta}_i)}{\sum_{j,k\in\{0,1\}} \pi_{jk}^{(t)} f_{jk}^{(t)}(\sqrt{n}\hat{\alpha}_i, \sqrt{n}\hat{\beta}_i)}, \quad u, v \in \{0,1\},
\end{aligned}$$

where $f_{uv}^{(t)}$ denotes the density evaluated using the parameters from iteration t. The expected complete data log-likelihood is then:

$$\begin{aligned}
\mathcal{Q}(\Gamma \mid \Gamma^{(t)}) = &\sum_{i=1}^m \sum_{u,v\in\{0,1\}} Q_{i;u,v}^{(t)} \log f_{uv}(\sqrt{n}\hat{\alpha}_i, \sqrt{n}\hat{\beta}_i) \\
&+ \sum_{i=1}^m \sum_{u,v\in\{0,1\}} Q_{i;u,v}^{(t)} \log \pi_{uv}.
\end{aligned}$$

M-Step

We maximize $\mathcal{Q}(\Gamma \mid \Gamma^{(t)})$ with respect to Γ to obtain updated estimates.
1. Updating Mixing Proportions (π): Maximizing with respect to π_{uv} under the constraint $\sum_{u,v} \pi_{uv} = 1$ yields the standard closed-form update:

$$\pi_{uv}^{(t+1)} = \frac{1}{m} \sum_{i=1}^{m} Q_{i;u,v}^{(t)}, \quad u, v \in \{0, 1\}. \tag{S2.3}$$

2. Updating Means (μ, θ): Setting the partial derivative with respect to μ to zero:

$$\frac{\partial \mathcal{Q}}{\partial \mu} = \sum_{i=1}^{m} Q_{i;1,0}^{(t)} \frac{\sqrt{n}\hat{\alpha}_i - \mu}{\sigma_{i1}^2 + \kappa^{(t)}} + \sum_{i=1}^{m} Q_{i;1,1}^{(t)} \frac{\sqrt{n}\hat{\alpha}_i - \mu}{\sigma_{i1}^2 + \kappa^{(t)}} = 0.$$

Solving for μ yields the weighted least squares estimator:

$$\mu^{(t+1)} = \sqrt{n} \frac{\sum_{i=1}^{m} w_i^{(t)} \hat{\alpha}_i}{\sum_{i=1}^{m} w_i^{(t)}}, \quad \text{where } w_i^{(t)} = \frac{Q_{i;1,0}^{(t)} + Q_{i;1,1}^{(t)}}{\sigma_{i1}^2 + \kappa^{(t)}}.$$

Similarly, maximizing with respect to θ yields:

$$\theta^{(t+1)} = \sqrt{n} \frac{\sum_{i=1}^{m} v_i^{(t)} \hat{\beta}_i}{\sum_{i=1}^{m} v_i^{(t)}}, \quad \text{where } v_i^{(t)} = \frac{Q_{i;0,1}^{(t)} + Q_{i;1,1}^{(t)}}{\sigma_{i2}^2 + \psi^{(t)}}.$$

3. Updating Variances (κ, ψ): Due to the heterogeneity of σ_{i1}^2 and σ_{i2}^2, closed-form updates for κ and ψ are not available. Therefore, the estimates $\kappa^{(t+1)}$ and $\psi^{(t+1)}$ are obtained by performing a 1-D numerical grid search on the likelihood function given in (S2.2).

C Two-Step EM Algorithm

Step 1: Marginal Parameter Estimation
In the first step, we fit a $(d_1 + 1)$-component univariate GMM to the statistics $\sqrt{n}\hat{\alpha}_i$:

$$\sqrt{n}\hat{\alpha}_i \sim \sum_{u=0}^{d_1} \pi_{u\cdot} N(\mu_u, \sigma_{i1}^2 + \kappa_u).$$

The posterior probability of component u given the i-th observation at iteration t is:

$$Q_{iu}^{(t)} = \frac{\pi_{u\cdot}^{(t)} \phi(\sqrt{n}\hat{\alpha}_i; \mu_u^{(t)}, \sigma_{i1}^2 + \kappa_u^{(t)})}{\sum_{k=0}^{d_1} \pi_{k\cdot}^{(t)} \phi(\sqrt{n}\hat{\alpha}_i; \mu_k^{(t)}, \sigma_{i1}^2 + \kappa_k^{(t)})}.$$

The mean parameters μ_u are updated via weighted least squares:

$$\mu_u^{(t+1)} = \sqrt{n} \frac{\sum_{i=1}^{m} w_{iu}^{(t)} \hat{\alpha}_i}{\sum_{i=1}^{m} w_{iu}^{(t)}}, \quad \text{where } w_{iu}^{(t)} = \frac{Q_{iu}^{(t)}}{\sigma_{i1}^2 + \kappa_u^{(t)}}.$$

The variance parameters κ_u are estimated via a 1-D grid search maximizing the marginal log-likelihood. The parameters $\{\theta_v, \psi_v\}_{v=1}^{d_2}$ corresponding to $\sqrt{n}\hat{\beta}_i$ are estimated analogously.
Step 2: Joint Mixing Proportion Estimation
We fix the parameters $\{\hat{\mu}_u, \hat{\kappa}_u\}_{u=1}^{d_1}$ and $\{\hat{\theta}_v, \hat{\psi}_v\}_{v=1}^{d_2}$ obtained from Step 1. Let the latent state be denoted by $\boldsymbol{\xi_i} = (\xi_{i1}, \xi_{i2})$, where $\xi_{i1} \in \{0, \dots, d_1\}$ and $\xi_{i2} \in \{0, \dots, d_2\}$.

Our goal is to estimate the joint probability matrix $\pi_{uv} = \mathbb{P}(\xi_{i1} = u, \xi_{i2} = v)$. Using the fixed marginal parameters, we compute the conditional probability of the latent state $\boldsymbol{\xi_i}$ given the data:

$$P_{i;u,v}^{(t)} = \frac{\pi_{uv}^{(t)} \phi(\sqrt{n}\hat{\alpha}_i, \sqrt{n}\hat{\beta}_i \mid \xi_{i1} = u, \xi_{i2} = v)}{\sum_{j=0}^{d_1} \sum_{k=0}^{d_2} \pi_{jk}^{(t)} \phi(\sqrt{n}\hat{\alpha}_i, \sqrt{n}\hat{\beta}_i \mid \xi_{i1} = j, \xi_{i2} = k)}.$$

The joint mixing proportions are updated as:

$$\pi_{uv}^{(t+1)} = \frac{1}{m} \sum_{i=1}^{m} P_{i;u,v}^{(t)}.$$

We iterate this procedure until convergence. Finally, the aggregate probabilities for the composite hypotheses are computed by summing over the relevant indices:

$$\begin{aligned}
\mathbb{P}(H_{00}) &= \pi_{00}, \\
\mathbb{P}(H_{10}) &= \sum_{u=1}^{d_1} \pi_{u0}, \\
\mathbb{P}(H_{01}) &= \sum_{v=1}^{d_2} \pi_{0v}, \\
\mathbb{P}(H_{11}) &= \sum_{u=1}^{d_1} \sum_{v=1}^{d_2} \pi_{uv}.
\end{aligned}$$

D Comparison: Standard EM vs. Two-Step EM

The two-step EM algorithm is designed primarily to mitigate the computational burden associated with increasing component counts (d_1, d_2). To evaluate the trade-offs between the standard bivariate EM (MLFDR) and the two-step variant (MLFDR-2), we conducted a simulation with $d_1 = d_2 = 1$. We considered sample sizes of $n = 100$ and 300 for $m = 1000$ under the Dense alternative scenarion, i.e $\boldsymbol{\pi} = \{0.4, 0.2, 0.2, 0.2\}$ The data were generated as follows:

$$\begin{aligned}
X &\sim \text{Ber}(0.1), \\
M_i &= \alpha_i X + e_i, \\
Y_i &= \beta_i M_i + \gamma_i X + \epsilon_i.
\end{aligned}$$

S1 Fig compares the empirical FDR and power of HDMT, DACT, MLFDR (using standard bivariate EM), and MLFDR-2 (using two-step EM).
We also benchmarked the computation times for comparable dimensions, scaling up to $m = 50,000$ and $n = 500$ (Table A). The results demonstrate that while the standard EM is efficient for smaller datasets, the two-step EM scales significantly better for high-dimensional data. For instance, at $m = 50,000$, MLFDR-2 is approximately five times faster than the standard MLFDR.

Method	$m = 1000$	$m = 5000$	$m = 50,000$
	$n = 100$	$n = 200$	$n = 500$
MLFDR	247 ms	1006 ms	6253 ms
MLFDR-2	1209 ms	1246 ms	1166 ms

Table A. Computation times (in milliseconds) for MLFDR vs. Two-Step MLFDR across varying dimensions.

As shown in S1 Fig, both MLFDR and MLFDR-2 offer power improvements over HDMT and DACT. However, the two-step approximation results in a reduction in power compared to the exact bivariate EM. Given that this marginal power loss could translate to missed discoveries in large-scale genomic studies, we recommend using the standard bivariate GMM when the model complexity is low (e.g., $d_1 + d_2 \leq 3$). For more complex models where computational resources are a constraint, the two-step EM provides a viable and efficient alternative.

E Theory

To establish the asymptotic properties of MLFDR, we first define the limiting random variables $(\alpha_{i,0}, \beta_{i,0})$. Conditional on the latent state $\boldsymbol{\xi}_i = (\xi_{i1}, \xi_{i2})$, these variables follow a Gaussian Mixture Model (GMM):

$$(\alpha_{i,0}, \beta_{i,0}) \sim N\left(\begin{pmatrix}\mu\xi_{i1}\\ \theta\xi_{i2}\end{pmatrix}, \begin{pmatrix}\sigma_{i1,0}^2 + \kappa\xi_{i1} & 0\\ 0 & \sigma_{i2,0}^2 + \psi\xi_{i2}\end{pmatrix}\right), \tag{S5.4}$$

where the limiting variances are defined as $\sigma_{i1,0}^2 = \sigma_{i,a}^2/\mathbb{E}[X^2]$ and $\sigma_{i2,0}^2 = \sigma_{i,b}^2\mathbb{E}[X^2]/\{\mathbb{E}[X^2]\mathbb{E}[M_i^2] - (\mathbb{E}[XM_i])^2\}$.
Let $G^0(t)$ and $G_{jk}^0(t)$ denote the cumulative distribution functions of the local FDR based on these limiting variables:

$$G^0(t) = \frac{1}{m}\sum_{i=1}^m \mathbb{P}(\mathrm{lfdr}(\alpha_{i,0}, \beta_{i,0}) \leq t),$$
$$G_{jk}^0(t) = \frac{1}{m}\sum_{i=1}^m \mathbb{P}(\mathrm{lfdr}(\alpha_{i,0}, \beta_{i,0}) \leq t \mid H_{jk}^0), \quad \text{for} \quad j,k \in \{0,1\}.$$

We further define the limiting function $V(\delta) := \pi_{00}G_{00}^0(\delta) + \pi_{10}G_{10}^0(\delta) + \pi_{01}G_{01}^0(\delta)$. Our theoretical analysis relies on establishing the following pointwise convergence results:

$$\frac{1}{m}V_m(\delta) \xrightarrow{P} V(\delta), \quad \frac{1}{m}W_m(\delta) \xrightarrow{P} V(\delta), \quad \frac{1}{m}R_m(\delta) \xrightarrow{P} G^0(\delta). \tag{S5.5}$$

Additionally, we define the theoretical and empirical marginal FDR (mFDR) processes as:

$$Q(\delta) = \frac{V(\delta)}{G^0(\delta)}, \quad \widetilde{Q}_m(\delta) = \frac{V_m(\delta)}{R_m(\delta) \vee 1}. \tag{S5.6}$$

Note that at a cutoff δ, the FDR for the oracle procedure is given by $\mathrm{FDR}_m(\delta) = \mathbb{E}[\widetilde{Q}_m(\delta)]$.
We assume the following conditions to prove asymptotic FDR control:
(A1) EM estimates are AMLE. We assume the estimators satisfy the Approximate Maximum Likelihood Estimator (AMLE) property. An estimator $\hat{f}$ is an AMLE of a density f based on samples $(\alpha_1, \beta_1), \ldots, (\alpha_m, \beta_m)$ if:

$$\prod_{i=1}^m \frac{\hat{f}(\alpha_i, \beta_i)}{f(\alpha_i, \beta_i)} \geq 1.$$

This condition, previously utilized by [2], ensures that the estimator yields a likelihood at least as high as the true parameters. This property is essential for establishing the convergence of the estimated GMM density to the truth under the Hellinger distance, thereby ensuring the identifiability of the EM algorithm.

(A2) Independence of samples. The observations $\{M_i^j\}_{j=1}^n$ are mutually independent, as are $\{X_j\}_{j=1}^n$.
(A3) Sub-Gaussianity. The variables satisfy the following tail conditions: $M_i \sim \text{subG}(\nu_i^2)$, $M_i X \sim \text{subG}(\eta_i^2)$, and $X \sim \text{subG}(\rho^2)$. Note that if $M_i \sim \text{subG}(\nu_i^2)$, it follows that $(M_i^2 - \mathbb{E}M_i^2) \sim \text{subE}(16\nu_i^2)$ and $(X^2 - \mathbb{E}X^2) \sim \text{subE}(16\rho^2)$, where subG and subE denote sub-Gaussian and sub-exponential variables, respectively. A variable Z is sub-Gaussian with parameter ρ if $\mathbb{P}(|Z| \geq t) \leq 2\exp(-\frac{t^2}{2\rho^2})$. A variable Y is sub-exponential with parameter η if

$$\mathbb{P}(|Y - \mathbb{E}Y| > t) \leq 2\exp\left(-\frac{1}{2}\min\left(\frac{t}{\eta}, \frac{t^2}{\eta^2}\right)\right).$$

(A4) Finite variance. There exist constants c, C such that $0 < c < \min_i \sigma_{ij,0}^2 \leq \max_i \sigma_{ij,0}^2 < C < \infty$ for $j = 1, 2$.
(A5) Critical point. There exists a $\delta_\infty \in (0, 1]$ such that $Q(\delta_\infty) < \alpha$.
Assumptions **(A2)**–**(A4)** enable the derivation of the uniform convergence of σ_{i1}^2 and σ_{i2}^2 to their limiting counterparts in (S5.4) (Lemma 1). This variance convergence is subsequently used to prove the pointwise convergence results in Lemma 2. Assumption **(A5)**, similar to conditions used in [1], ensures the existence of a valid cutoff that asymptotically controls the FDR at level α.
We state the main theoretical results below. Theorem 1 establishes FDR control for the oracle rule, while Theorem 2 extends this control to the adaptive procedure.

Theorem 1. *Under Assumptions **(A2)** – **(A5)**, if $m, n \to \infty$ such that $m = o(\exp(kn))$ for some constant k, then:*

$$\limsup_{m\to\infty} FDR_m(\delta_m) \leq \alpha.$$

Theorem 2. *Under Assumptions **(A1)** – **(A5)**, if $m, n \to \infty$ such that $m = o(\exp(kn))$ for some constant k, then:*

$$\limsup_{m\to\infty} FDR_m(\widehat{\delta}_m) \leq \alpha.$$

To prove Theorem 1 and Theorem 2 we first establish a few ancillary results.

Lemma 1. *Under Assumptions **(A2)** - **(A4)**, if $m, n \to \infty$ such that $m = o(exp(kn))$ for all $0 < k < \infty$, then*

$$\max_{1\leq i\leq m} |\sigma_{i1}^2 - \sigma_{i1,0}^2| \to^p 0,$$

and

$$\max_{1\leq i\leq m} |\sigma_{i2}^2 - \sigma_{i2,0}^2| \to^p 0.$$

Proof. Define $D_n := n^{-1}\sum_{j=1}^n (X^j)^2$ and $D := \mathbb{E}[X^2]$. Since $D > 0$, there exists a real number a such that $D > 2a > 0$. Under Assumption **(A3)**, X is sub-Gaussian, which implies that X^2 is sub-exponential. Applying Bernstein's inequality to the centered

term $D_n - D$, we have:

$$\begin{aligned}\sum_{i=1}^{m} \mathbb{P}\left(\left|\sigma_{i1}^2 - \sigma_{i1,0}^2\right| > \epsilon\right) &= \sum_{i=1}^{m} \mathbb{P}\left(\left|\frac{1}{D_n} - \frac{1}{D}\right| > \delta_i\right) \\ &\leq \sum_{i=1}^{m} \mathbb{P}\left(\left|\frac{1}{D_n} - \frac{1}{D}\right| > \delta_i, D_n > a\right) + \sum_{i=1}^{m} \mathbb{P}(D_n \leq a) \\ &\leq \sum_{i=1}^{m} \mathbb{P}\left(|D_n - D| > 2a^2\delta_i\right) + \sum_{i=1}^{m} \mathbb{P}(|D_n - D| \geq a) \\ &\leq 2\sum_{i=1}^{m} \exp\left(-\frac{n}{2}\left(\frac{4a^4\delta_i^2}{256\rho^4} \wedge \frac{2a^2\delta_i}{16\rho^2}\right)\right) \\ &\quad + 2m\exp\left(-\frac{n}{2}\left(\frac{a^2}{256\rho^4} \wedge \frac{a}{16\rho^2}\right)\right),\end{aligned}$$

where $\delta_i = \epsilon/\sigma_{i,a}^2$.

Next, we consider the convergence of the mediator moments. By Assumption **(A3)**, M_i^2 is sub-exponential. Using Bernstein's inequality and the union bound, we obtain:

$$\begin{aligned}\mathbb{P}\left(\max_{1\leq i\leq m}\left|n^{-1}\sum_{j=1}^{n}(M_i^j)^2 - \mathbb{E}[M_i^2]\right| > \epsilon\right) &\leq \sum_{i=1}^{m}\mathbb{P}\left(\left|n^{-1}\sum_{j=1}^{n}(M_i^j)^2 - \mathbb{E}[M_i^2]\right| > \epsilon\right) \\ &\leq 2\sum_{i=1}^{m}\exp\left(-\frac{n}{2}\left(\frac{\epsilon^2}{256\nu_i^4} \wedge \frac{\epsilon}{16\nu_i^2}\right)\right) \\ &\to 0.\end{aligned}$$

Similarly, since M_iX is sub-Gaussian by assumption, we apply Chernoff's bound and the union bound:

$$\begin{aligned}\mathbb{P}\left(\max_{1\leq i\leq m}\left|n^{-1}\sum_{j=1}^{n}M_i^jX^j - \mathbb{E}[M_iX]\right| > \epsilon\right) &\leq 2\sum_{i=1}^{m}\exp\left(-\frac{n\epsilon^2}{2\eta_i^2}\right) \\ &\to 0.\end{aligned}$$

To analyze σ_{i2}^2, we introduce the following notation:

$$\begin{aligned}A_{n,i} &:= \frac{1}{n}\sum_{j=1}^{n}(M_i^j)^2, & A_i &:= \mathbb{E}[M_i^2], \\ B_{n,i} &:= \frac{1}{n}\sum_{j=1}^{n}M_i^jX^j, & B_i &:= \mathbb{E}[M_iX], \\ C_n &:= \frac{1}{n}\sum_{j=1}^{n}X^j, & C &:= \mathbb{E}[X].\end{aligned}$$

For a fixed i, the estimator σ_{i2}^2 is a continuous function of $(A_{n,i}, B_{n,i}, C_n)$ at the point

(A_i, B_i, C). By the definition of continuity, for any $\epsilon > 0$, there exists a $\delta > 0$ such that:

$$
\begin{aligned}
\mathbb{P}\left(\max_{1\le i\le m} |\sigma_{i2}^2 - \sigma_{i2,0}^2| > \epsilon\right) &< \sum_{i=1}^{m} \mathbb{P}(|\sigma_{i2}^2 - \sigma_{i2,0}^2| > \epsilon) \\
&\le \sum_{i=1}^{m} \mathbb{P}\left((A_{n,i} - A_i)^2 + (B_{n,i} - B_i)^2 + (C_n - C)^2 > \delta^2\right) \\
&\le \sum_{i=1}^{m} \Big\{\mathbb{P}(|A_{n,i} - A_i| > \delta/\sqrt{3}) + \mathbb{P}(|B_{n,i} - B_i| > \delta/\sqrt{3}) \\
&\quad + \mathbb{P}(|C_n - C| > \delta/\sqrt{3})\Big\} \\
&\le 2\sum_{i=1}^{m} \exp\left(-\frac{n}{2}\left(\frac{\delta^2/3}{256\nu_i^4} \wedge \frac{\delta/\sqrt{3}}{16\nu_i^2}\right)\right) \\
&\quad + 2\sum_{i=1}^{m} \exp\left(-\frac{n\delta^2}{6\eta_i^2}\right) + m\exp\left(-\frac{n\delta^2}{6\rho^2}\right) \\
&\to 0.
\end{aligned}
$$

Therefore, we conclude that:

$$\max_{1\le i\le m} |\sigma_{i2}^2 - \sigma_{i2,0}^2| \xrightarrow{p} 0. \tag{S5.7}$$

□

Lemma 2. *Let V_m, W_m, R_m be defined as in Equations (14), (15), and (16). As $m, n \to \infty$, the following convergence results hold:*

$$\frac{1}{m} V_m(\delta) \xrightarrow{P} V(\delta), \tag{S5.8}$$

$$\frac{1}{m} W_m(\delta) \xrightarrow{P} V(\delta), \tag{S5.9}$$

$$\frac{1}{m} R_m(\delta) \xrightarrow{P} G^0(\delta). \tag{S5.10}$$

Proof. Conditional on X and $\{M_i\}_i$, the local FDR statistics $\mathrm{lfdr}(\sqrt{n}\hat{\alpha}_i, \sqrt{n}\hat{\beta}_i)$ are independent across i. Applying the Dvoretzky–Kiefer–Wolfowitz inequality [4], we have:

$$
\begin{aligned}
&\mathbb{P}\left(\sup_{\delta}\left|\frac{V_m(\delta) - \sum_{i\in H_0}\mathbb{P}(\mathrm{lfdr}(\sqrt{n}\hat{\alpha}_i, \sqrt{n}\hat{\beta}_i) \le \delta \mid X, \{M_i\}_i, H_{0,i})}{m}\right| > \epsilon \;\middle|\; X, \{M_i\}_i\right) \\
&\quad \le d(m+1)\exp(-2m\epsilon^2).
\end{aligned}
$$

Unconditioning yields the unconditional bound:

$$
\begin{aligned}
&\mathbb{P}\left(\sup_{\delta}\left|\frac{V_m(\delta) - \sum_{i\in H_0}\mathbb{P}(\mathrm{lfdr}(\sqrt{n}\hat{\alpha}_i, \sqrt{n}\hat{\beta}_i) \le \delta \mid X, \{M_i\}_i, H_{0,i})}{m}\right| > \epsilon\right) \\
&\quad \le d(m+1)\exp(-2m\epsilon^2).
\end{aligned}
$$

Define the conditional cumulative distribution function:

$$K_t(\sigma_{i1}^2, \sigma_{i2}^2) = \mathbb{P}(\mathrm{lfdr}(\sqrt{n}\hat{\alpha}_i, \sqrt{n}\hat{\beta}_i) \le t \mid X, \{M_i\}_i, H_{00}).$$

Since the distribution of $(\sqrt{n}\hat{\alpha}_i, \sqrt{n}\hat{\beta}_i)$ given $X, \{M_i\}_i$ differs from the limiting distribution $(\alpha_{i,0}, \beta_{i,0})$ only via the variances—$(\sigma_{i1}^2, \sigma_{i2}^2)$ versus $(\sigma_{i1,0}^2, \sigma_{i2,0}^2)$—we can identify:

$$K_t(\sigma_{i1,0}^2, \sigma_{i2,0}^2) = \mathbb{P}(\text{lfdr}(\alpha_{i,0}, \beta_{i,0}) \le t \mid H_{00}^0).$$

The function $K_t(a, b)$ is continuous with respect to (a, b). Therefore, applying the continuous mapping theorem and the convergence results from Lemma 1, we obtain:

$$\begin{aligned}
&\mathbb{P}(|G_{00}(t) - G_{00}^0(t)| > \epsilon) \\
&\le \mathbb{P}\left(\sum_{i=1}^{m} |K_t(\sigma_{i1}^2, \sigma_{i2}^2) - K_t(\sigma_{i1,0}^2, \sigma_{i2,0}^2)| > \epsilon m\right) \\
&\le \sum_{i=1}^{m} \mathbb{P}(|K_t(\sigma_{i1}^2, \sigma_{i2}^2) - K_t(\sigma_{i1,0}^2, \sigma_{i2,0}^2)| > \epsilon) \\
&\le \sum_{i=1}^{m} \mathbb{P}\left(|\sigma_{i1}^2 - \sigma_{i1,0}^2|^2 + |\sigma_{i2}^2 - \sigma_{i2,0}^2|^2 > \delta^2\right) \\
&\le \sum_{i=1}^{m} \left[\mathbb{P}\left(|\sigma_{i1}^2 - \sigma_{i1,0}^2| > \frac{\delta}{\sqrt{2}}\right) + \mathbb{P}\left(|\sigma_{i2}^2 - \sigma_{i2,0}^2| > \frac{\delta}{\sqrt{2}}\right)\right] \\
&\to 0.
\end{aligned}$$

By analogous arguments, we establish:

$$\begin{aligned}
G_{10}(t) &\xrightarrow{P} G_{10}^0(t), \\
G_{01}(t) &\xrightarrow{P} G_{01}^0(t), \\
G_{11}(t) &\xrightarrow{P} G_{11}^0(t).
\end{aligned}$$

Combining these convergence results, it follows that:

$$\frac{1}{m} \sum_{i \in H_0} \mathbb{P}(\text{lfdr}(\sqrt{n}\hat{\alpha}_i, \sqrt{n}\hat{\beta}_i) \le \delta \mid X, \{M_i\}_i, H_{0,i}) \xrightarrow{P} V(\delta). \tag{S5.11}$$

The convergence of $W_m(\delta)$ and $R_m(\delta)$ follows a similar derivation. □

Proof of Theorem 1. Invoking the Glivenko-Cantelli theorem, we establish the following uniform convergence results:

$$\begin{aligned}
\sup_{\delta \in [0,1]} \left|\frac{1}{m} R_m(\delta) - G^0(\delta)\right| &\xrightarrow{P} 0, \\
\sup_{\delta \in [0,1]} \left|\frac{1}{m} V_m(\delta) - V(\delta)\right| &\xrightarrow{P} 0, \\
\sup_{\delta \in [0,1]} \left|\frac{1}{m} W_m(\delta) - V(\delta)\right| &\xrightarrow{P} 0.
\end{aligned} \tag{S5.12}$$

Define the realized False Discovery Proportion (FDP) process as:

$$\widetilde{Q}_m(\delta) = \frac{V_m(\delta)}{R_m(\delta)}.$$

Following arguments analogous to Lemma 8.2 in [1], we have:

$$\begin{aligned}
\sup_{\delta \ge \delta_\infty} |Q_m(\delta) - Q(\delta)| &\xrightarrow{P} 0, \\
\sup_{\delta \ge \delta_\infty} \left|\widetilde{Q}_m(\delta) - Q(\delta)\right| &\xrightarrow{P} 0.
\end{aligned} \tag{S5.13}$$

The remainder of the proof follows the logic of Proposition 2.2 in [1]. Define $e = \alpha - Q(\delta_\infty)$. By Assumption **(A5)**, we have $e > 0$. Combining this with (S5.13), implies that $\mathbb{P}(|Q_m(\delta_\infty) - Q(\delta_\infty)| < e/2) \to 1$. Consequently, $\mathbb{P}(Q_m(\delta_\infty) < \alpha) \to 1$. By the definition of δ_m, this implies that $\mathbb{P}(\delta_m \geq \delta_\infty) \to 1$.
We then bound the difference between the estimated and realized FDP at the threshold δ_m:

$$\begin{aligned} Q_m(\delta_m) - \widetilde{Q}_m(\delta_m) &\geq \inf_{\delta \geq \delta_\infty} \{Q_m(\delta) - \widetilde{Q}_m(\delta)\} \\ &\geq \inf_{\delta \geq \delta_\infty} \{Q_m(\delta) - Q(\delta) + Q(\delta) - \widetilde{Q}_m(\delta)\} \\ &= o_{\mathbb{P}}(1). \end{aligned}$$

Therefore,

$$\widetilde{Q}_m(\delta_m) \leq Q_m(\delta_m) + o_{\mathbb{P}}(1) \leq \alpha + o_{\mathbb{P}}(1).$$

This implies:

$$\frac{V_m(\delta_m)}{R_m(\delta_m) \vee 1} \leq \frac{V_m(\delta_m)}{R_m(\delta_m)} = \widetilde{Q}_m(\delta_m) \leq \alpha + o_{\mathbb{P}}(1).$$

Finally, applying Lemma 8.3 of [1] ensures uniform integrability, yielding:

$$\limsup_{m \to \infty} \mathrm{FDR}_m(\delta_m) = \limsup_{m \to \infty} \mathbb{E}\left[\frac{V_m(\delta_m)}{R_m(\delta_m) \vee 1}\right] \leq \alpha.$$

□

Proof of Theorem 2. We define the empirical processes based on the estimated local FDR as:

$$\widehat{R}_m(\delta) := \sum_{i=1}^{m} \mathbf{1}\{\widehat{\mathrm{lfdr}}(\sqrt{n}\hat{\alpha}_i, \sqrt{n}\hat{\beta}_i) \leq \delta\}, \tag{S5.14}$$

$$\widehat{W}_m(\delta) := \sum_{i=1}^{m} \mathbf{1}\{\widehat{\mathrm{lfdr}}(\sqrt{n}\hat{\alpha}_i, \sqrt{n}\hat{\beta}_i) \leq \delta\}\widehat{\mathrm{lfdr}}(\sqrt{n}\hat{\alpha}_i, \sqrt{n}\hat{\beta}_i). \tag{S5.15}$$

Following the argument in Lemma 8.4 of [1] and invoking Lemma 5, we establish the uniform convergence:

$$\sup_{\delta \geq \delta_\infty} \left| \frac{1}{m}\widehat{R}_m(\delta) - G^0(\delta) \right| \xrightarrow{P} 0, \tag{S5.16}$$

$$\sup_{\delta \geq \delta_\infty} \left| \frac{1}{m}\widehat{W}_m(\delta) - V(\delta) \right| \xrightarrow{P} 0. \tag{S5.17}$$

Using arguments similar to Lemma 8.2 of [1], it follows that:

$$\sup_{\delta \geq \delta_\infty} \left| \widehat{Q}_m(\delta) - Q(\delta) \right| \xrightarrow{P} 0.$$

Proceeding analogously to Theorem 1, set $e = \alpha - Q(\delta_\infty) > 0$. We have:

$$\left| \widehat{Q}_m(\delta_\infty) - Q(\delta_\infty) \right| \leq \sup_{\delta \geq \delta_\infty} \left| \widehat{Q}_m(\delta) - Q(\delta) \right| \leq e/2 \quad \text{with probability } \to 1.$$

This implies $\mathbb{P}(\widehat{Q}_m(\delta_\infty) < \alpha) \to 1$, and consequently $\mathbb{P}(\widehat{\delta}_m \geq \delta_\infty) \to 1$. Conditional on the event $\{\widehat{\delta}_m \geq \delta_\infty\}$, we have:

$$\begin{aligned}
\widetilde{Q}_m(\widehat{\delta}_m) - \widehat{Q}_m(\widehat{\delta}_m) &\leq \sup_{\delta \geq \delta_\infty} \left|\widehat{Q}_m(\delta) - \widetilde{Q}_m(\delta)\right| \\
&\leq \sup_{\delta \geq \delta_\infty} \left|\widehat{Q}_m(\delta) - Q(\delta)\right| + \sup_{\delta \geq \delta_\infty} \left|Q(\delta) - \widetilde{Q}_m(\delta)\right| \\
&= o_{\mathbb{P}}(1).
\end{aligned}$$

By the definition of $\widehat{\delta}_m$, we have $\widehat{Q}_m(\widehat{\delta}_m) \leq \alpha$. Therefore:

$$\frac{V_m(\widehat{\delta}_m)}{R_m(\widehat{\delta}_m) \vee 1} \leq \frac{V_m(\widehat{\delta}_m)}{R_m(\widehat{\delta}_m)} = \widetilde{Q}_m(\widehat{\delta}_m) \leq \alpha + o_{\mathbb{P}}(1).$$

Applying Lemma 8.3 of [1], we conclude:

$$\limsup_{m\to\infty} \mathrm{FDR}_m(\widehat{\delta}_m) = \limsup_{m\to\infty} \mathbb{E}\left[\frac{V_m(\widehat{\delta}_m)}{R_m(\widehat{\delta}_m) \vee 1}\right] \leq \alpha.$$

□

Definition 3 (Gaussian Mixture Models)**.** Let $\mathcal{F}^2_{\text{Gauss}}$ denote the class of bivariate Gaussian Mixture Model (GMM) densities, defined as:

$$\mathcal{F}^2_{\text{Gauss}} = \left\{ f : f(\alpha, \beta) = \int \phi(\alpha, \beta; \mu, \theta, \sigma_1^2, \sigma_2^2, \rho)\, dG^*(\mu, \theta, \sigma_1^2, \sigma_2^2, \rho) \right\},$$

where G^* is a discrete probability measure supported on the set:

$$\begin{aligned}
&\mu \in [-M_1, M_1], \\
&\theta \in [-M_2, M_2], \\
&\sigma_1^2, \sigma_2^2 \in (c, C), \\
&\rho \in (0, 1).
\end{aligned}$$

We extend this definition to the class of b-variate GMM densities, denoted by $\mathcal{F}^b_{\text{Gauss}}$. Let G^d_b represent the corresponding mixing distribution with at most d atoms, defined on a bounded support. Specifically, if $\boldsymbol{\mu}_i$ and $\boldsymbol{\Sigma}_i = (\sigma^i_{kl})^b_{k,l=1}$ denote the mean vector and the covariance matrix of the i-th component for $i = 1, \ldots, d$, we assume:

$$\begin{aligned}
\boldsymbol{\mu}_i &\in [-M\mathbf{1}, M\mathbf{1}], \\
\sigma^i_{kk} &\in [c, C].
\end{aligned}$$

Note that this condition implies $\sigma^i_{kl} \in [-C, C]$ for $k \neq l$. Based on these parameters, we define G^d_b as:

$$G^d_b = \sum_{i=1}^d \pi_i \delta(\boldsymbol{\mu}_i, \boldsymbol{\Sigma}_i).$$

If $\phi(\mathbf{x}, \boldsymbol{\mu}, \boldsymbol{\Sigma})$ denotes the density of a b-dimensional multivariate normal distribution with mean $\boldsymbol{\mu}$ and covariance matrix $\boldsymbol{\Sigma}$, then:

$$\mathcal{F}^b_{\text{Gauss}} = \left\{ f : f(\mathbf{x}) = \sum_{i=1}^d \pi_i \phi(\mathbf{x}, \boldsymbol{\mu}_i, \boldsymbol{\Sigma}_i) = \int \phi(\mathbf{x}, \boldsymbol{\mu}, \boldsymbol{\Sigma})\, dG^d_b(\boldsymbol{\mu}, \boldsymbol{\Sigma}) \right\}.$$

Definition 4 (AMLE)**.** Let $\mathbf{X}$ be a d-dimensional random variable with density function $f : \mathbb{R}^d \to \mathbb{R}$ (or probability mass function for count data), where the parametric form of f may be unknown. Based on a sample $(\mathbf{X}_1, \mathbf{X}_2, \ldots, \mathbf{X}_m)$, an estimator $\hat{f}$ is defined as an Approximate Maximum Likelihood Estimator (AMLE) of f if:

$$\prod_{i=1}^{m} \frac{\hat{f}(\mathbf{X}_i)}{f(\mathbf{X}_i)} \geq 1.$$

That is, to qualify as an AMLE, the estimator must yield a likelihood evaluated at the observed samples that is at least as high as that of the true density.

Definition 5 (Hellinger Distance)**.** We quantify the discrepancy between the estimated density $\hat{f}$ and the true density f using the average squared Hellinger distance, given by:

$$\mathcal{D}(\hat{f}, f) = \frac{1}{m} \sum_{i=1}^{m} h^2(\hat{f}(\alpha_i, \beta_i), f(\alpha_i, \beta_i)),$$

where

$$h^2(\hat{f}(\alpha_i, \beta_i), f(\alpha_i, \beta_i)) = \frac{1}{2} \int \left(\sqrt{\hat{f}(\alpha_i, \beta_i)} - \sqrt{f(\alpha_i, \beta_i)} \right)^2 d\alpha_i d\beta_i.$$

Definition 6 (Total Variation Distance)**.** Alternatively, the loss between the estimated and true distributions can be quantified using the average Total Variation (TV) distance:

$$TV(\hat{f}, f) = \frac{1}{m} \sum_{i=1}^{m} \Delta(\hat{f}(\alpha_i, \beta_i), f(\alpha_i, \beta_i)),$$

where

$$\Delta(\hat{f}(\alpha_i, \beta_i), f(\alpha_i, \beta_i)) = \frac{1}{2} \int \left| \hat{f}(\alpha_i, \beta_i) - f(\alpha_i, \beta_i) \right| d\alpha_i d\beta_i.$$

Note that the Hellinger and Total Variation distances satisfy the inequality:

$$\mathcal{D}(\hat{f}, f) \leq TV(\hat{f}, f) \leq \sqrt{2}\sqrt{\mathcal{D}(\hat{f}, f)}.$$

Definition 7 (Restricted Supremum Norm)**.** Let $h, \tilde{h} : \mathbb{R}^d \to \mathbb{R}$ be two functions. We define the supremum norm over a bounded subset $S \subset \mathbb{R}^d$ as:

$$\left\| h - \tilde{h} \right\|_{\infty, S} := \sup_{\mathbf{x} \in S} \left| h(\mathbf{x}) - \tilde{h}(\mathbf{x}) \right|. \tag{S5.18}$$

Lemma 3. *Consider the family of b-variate Gaussian mixture distributions $\mathcal{F}^b_{Gauss}$ parameterized by the discrete mixing distributions G^d_b. Let $N(\eta, \mathcal{F}^b_{Gauss}, \|\cdot\|_{\infty,S})$ denote the covering number of this class under the restricted supremum norm defined in Equation (S5.18). Assuming that the covariance matrices of the component distributions are full-rank, the following bound holds:*

$$N(\eta, \mathcal{F}^b_{Gauss}, \|\cdot\|_{\infty,S}) \leq K \eta^{-\frac{2d+3db+db^2}{2}},$$

where K is a constant depending on the parameters (d, b, M, c, C).

Proof. Let $f_{G^d_b} \in \mathcal{F}^b_{\text{Gauss}}$ be a density function with mixing distribution $G^d_b = \sum_{i=1}^{d} \pi_i \delta(\boldsymbol{\mu}_i, \mathbf{S}_i)$, given by:

$$f_{G^d_b}(\mathbf{x}) = \sum_{i=1}^{d} \pi_i \phi(\mathbf{x}, \boldsymbol{\mu}_i, \mathbf{S}_i).$$

We assume the supports of the mean vectors $\boldsymbol{\mu}_i$ and covariance matrices $\mathbf{S}_i$ are bounded. We approximate $f_{G_b^d}$ by a discretized density $f_{G_{b,\eta}^d}$ supported on a lattice with at most d atoms. The approximating mixing distribution is:

$$G_{b,\eta}^d = \sum_{i=1}^{d} \pi_i \delta(\boldsymbol{\mu}_i^\eta, \mathbf{S}_i^\eta),$$

with corresponding density $f_{G_{b,\eta}^d}(\mathbf{x}) = \sum_{i=1}^{d} \pi_i \phi(\mathbf{x}, \boldsymbol{\mu}_i^\eta, \mathbf{S}_i^\eta)$.

Step 1: Discretization of Parameters The discretized parameters $\boldsymbol{\mu}_i^\eta$ and $\mathbf{S}_i^\eta = (\sigma_{i;kl}^\eta)_{k,l=1}^b$ are defined as:

$$\boldsymbol{\mu}_i^\eta = \eta \operatorname{sgn}(\boldsymbol{\mu}_i) \odot \left\lfloor \frac{|\boldsymbol{\mu}_i|}{\eta} \right\rfloor, \quad i = 1 \dots d, \tag{S5.19}$$

$$\sigma_{i;kl}^\eta = \eta \operatorname{sgn}(\sigma_{i;kl}) \left\lfloor \frac{|\sigma_{i;kl}|}{\eta} \right\rfloor, \quad k \neq l, \quad i = 1 \dots d, \tag{S5.20}$$

$$\sigma_{i;kk}^\eta = \eta \left\lfloor \frac{\sigma_{i;kk}}{\eta} \right\rfloor, \quad k = 1 \dots b. \tag{S5.21}$$

Define the parameter vector for the i-th component as $\mathcal{B}_i = (\boldsymbol{\mu}_i, \operatorname{vec}(\mathbf{S}_i))$. Then, for the Euclidean norm, we have:

$$\|\mathcal{B}_i - \mathcal{B}_{i;\eta}\| \leq \frac{3b + b^2}{2}\eta \quad \forall i = 1 \dots d.$$

Step 2: Bounding the Density Approximation Error We bound the supremum norm between the component densities using the Mean Value Theorem. Dropping the index i for brevity:

$$\|\phi(\mathbf{x}, \boldsymbol{\mu}, \mathbf{S}) - \phi(\mathbf{x}, \boldsymbol{\mu}^\eta, \mathbf{S}^\eta)\|_\infty \leq C_1 \|\mathcal{B} - \mathcal{B}_\eta\| \leq C_1 \frac{3b + b^2}{2}\eta,$$

where $C_1 = \sup_{\mathbf{x}} \sup_{\mathcal{B}} \|\nabla_{\mathcal{B}} \phi(\mathbf{x}, \boldsymbol{\mu}, \mathbf{S})\|$. The gradients are given by:

$$\frac{\partial \phi}{\partial \boldsymbol{\mu}} = \phi(\mathbf{x}, \boldsymbol{\mu}, \mathbf{S})\mathbf{S}^{-1}(\mathbf{x} - \boldsymbol{\mu}),$$
$$\frac{\partial \phi}{\partial \mathbf{S}} = T_1 + T_2,$$

where $T_1 = -\frac{1}{2}\mathbf{S}^{-1}\phi$ and $T_2 = \frac{1}{2}\mathbf{S}^{-1}(\mathbf{x} - \boldsymbol{\mu})(\mathbf{x} - \boldsymbol{\mu})'\mathbf{S}^{-1}\phi$.

We bound these terms using spectral theory. Assuming $\mathbf{S}$ is full rank, let λ_b and $\lambda_b^{(1/2)}$ be the smallest eigenvalues of $\mathbf{S}$ and $\mathbf{S}^{1/2}$ respectively. The Frobenius norm satisfies $\left\|\mathbf{S}^{-1}\right\|_F \leq \sqrt{b}/\lambda_b$. Noting that $\left\|\mathbf{S}^{-1/2}(\mathbf{x} - \boldsymbol{\mu})\phi\right\| \leq (2\pi)^{-b/2}\sqrt{e}$, we obtain:

$$\left\|\frac{\partial \phi}{\partial \boldsymbol{\mu}}\right\| \leq \left\|\mathbf{S}^{-1/2}\right\|_F \left\|\mathbf{S}^{-1/2}(\mathbf{x} - \boldsymbol{\mu})\phi\right\| \leq \frac{\sqrt{be}}{(2\pi)^{b/2}\lambda_b^{(1/2)}} := K_1.$$

For the covariance gradient:

$$\begin{aligned}
\left\|\frac{\partial \phi}{\partial \mathbf{S}}\right\|_F &\leq \|T_1\|_F + \|T_2\|_F \\
&\leq \frac{\sqrt{b}}{2(2\pi)^{b/2}\lambda_b} + \frac{b}{(\lambda_b^{(1/2)})^2} \left\|\frac{1}{(2\pi)^{b/2}} \mathbf{z}\mathbf{z}' e^{-\mathbf{z}'\mathbf{z}/2}\right\|_F \\
&\leq \frac{\sqrt{b}}{2(2\pi)^{b/2}\lambda_b} + \frac{be^{-0.25}}{2(\lambda_b^{(1/2)})^2(2\pi)^{b/2}} := K_2,
\end{aligned}$$

where $\mathbf{z} = \mathbf{S}^{-1/2}(\mathbf{x} - \boldsymbol{\mu})$. Summing over all d components:

$$\left\| f_{G_b^d} - f_{G_{b,\eta}^d} \right\|_\infty \leq d(K_1 + K_2)\frac{(3b + b^2)}{2}\eta := K_3\eta. \tag{S5.22}$$

Step 3: Covering Number Calculation The parameters are supported on grids Ω_1 (means), Ω_2 (off-diagonal covariances), and Ω_3 (diagonal covariances) with cardinalities:

$$|\Omega_1| \approx \frac{2M}{\eta}, \quad |\Omega_2| \approx \frac{2C}{\eta}, \quad |\Omega_3| \approx \frac{C^2}{\eta}.$$

Let $\mathcal{P}^{d,\eta}$ be an η-net for the mixing weights simplex $\mathcal{P}^d$. By the volume comparison lemma, $|\mathcal{P}^{d,\eta}| \leq (1 + 2/\eta)^d$. Choosing weights $\mathbf{w}^{d,\eta} \in \mathcal{P}^{d,\eta}$ such that $\sum |G_{b,\eta}^d - w_i^{d,\eta}| \leq \eta$, the error contribution from weights is bounded by $(2\pi)^{-b/2}\eta$. Combining the errors, the total approximation error is bounded by $K_4\eta$. The total covering number is the product of the number of choices for weights, means, diagonal variances, and off-diagonal covariances:

$$N(\eta, \mathcal{F}_{\text{Gauss}}^b, \|.\|_\infty) \leq |\mathcal{P}^{d,\eta}| \cdot |\Omega_1|^{db} \cdot |\Omega_3|^{db} \cdot |\Omega_2|^{\frac{db(b-1)}{2}}. \tag{S5.23}$$

Substituting the grid sizes and simplifying for $\eta \to 0$:

$$\begin{aligned} N(\eta, \mathcal{F}_{\text{Gauss}}^b, \|.\|_\infty) &\leq K' \left(\frac{1}{\eta}\right)^d \left(\frac{1}{\eta}\right)^{db} \left(\frac{1}{\eta}\right)^{db} \left(\frac{1}{\eta}\right)^{\frac{db(b-1)}{2}} \\ &= K\eta^{-\left(d+2db+\frac{db^2-db}{2}\right)} \\ &= K\eta^{-\frac{2d+3db+db^2}{2}}. \end{aligned}$$

□

Lemma 4. *Suppose* $(\alpha_1, \beta_1), \ldots, (\alpha_m, \beta_m)$ *are samples drawn from* $\mathcal{F}_{Gauss}^2$*. Given an AMLE* $\hat{f}$ *of* f*, we have for* $t \geq 1$ *and* $\delta > 0$*:*

$$\mathbb{P}\Big\{\mathcal{D}(\hat{f}, f) \geq t\delta\Big\} \leq C^* \log m \exp\left(20.31t^2(M^*)^2 - \frac{mt^2\delta^2}{2}\right) + 2m^{-\frac{t^2}{C}}.$$

Proof. Define the bounded rectangular region $S = [-M_1 - 2t\sqrt{\log m}, M_1 + 2t\sqrt{\log m}] \times [-M_2 - 2t\sqrt{\log m}, M_2 + 2t\sqrt{\log m}]$ and the event $A_t = \bigcap_{i=1}^m \big\{(\alpha_i, \beta_i) \in S\big\}$.

Part 1: Bounding $\mathbb{P}(A_t^c)$

We first show that $\mathbb{P}(A_t) \geq 1 - 2m^{-t^2/C}$. By the union bound:

$$\mathbb{P}(A_t^c) \leq \mathbb{P}(|\alpha_i| > M_1 + 2t\sqrt{\log m}) + \mathbb{P}(|\beta_i| > M_2 + 2t\sqrt{\log m}).$$

Since $(\alpha_i, \beta_i) \sim \mathcal{F}_{\text{Gauss}}^2$, we have $\alpha_i = \mu_i + \sigma_{i1}Z_{i1}$ and $\beta_i = \theta_i + \sigma_{i2}Z_{i2}$, where (Z_{i1}, Z_{i2}) are standard normals. The means μ_i and θ_i are supported on $[-M_1, M_1]$ and $[-M_2, M_2]$ respectively. Thus, for $t \geq 1, m \geq 2$:

$$\begin{aligned} \mathbb{P}(|\alpha_i| > M_1 + 2t\sqrt{\log m}) &= \mathbb{P}(|\mu_i + \sigma_{i1}Z_{i1}| > M_1 + 2t\sqrt{\log m}) \\ &\leq \mathbb{P}(\sigma_{i1}|Z_{i1}| > 2t\sqrt{\log m}) \\ &= 2\left(1 - \Phi\left(\frac{2t\sqrt{\log m}}{\sigma_{i1}}\right)\right) \\ &\leq 2\left(1 - \Phi\left(\frac{2t\sqrt{\log m}}{\sqrt{C}}\right)\right). \end{aligned}$$

Using the inequality $1 - \Phi(x) \leq \frac{\phi(x)}{x}$ for $x > 0$:

$$\begin{aligned}
\mathbb{P}(|\alpha_i| > M_1 + 2t\sqrt{\log m}) &\leq \frac{2\sqrt{C}}{2t\sqrt{\log m}} \cdot \frac{1}{\sqrt{2\pi}} \exp\left(-\frac{1}{2}\frac{4t^2 \log m}{C}\right) \\
&= \frac{\sqrt{C}}{t\sqrt{2\pi \log m}} m^{-2t^2/C} \\
&\leq m^{-t^2/C}.
\end{aligned}$$

Similarly, $\mathbb{P}(|\beta_i| > M_2 + 2t\sqrt{\log m}) \leq m^{-t^2/C}$. Therefore:

$$\mathbb{P}(A_t^c) \leq 2m^{-t^2/C}. \tag{S5.24}$$

Part 2: Bounding the Hellinger Distance

We now bound $\mathbb{P}\left\{\mathcal{D}(\hat{f}, f) \geq t\delta \cap A_t\right\}$. Let $\eta = m^{-2}$ and let $\{h_1, \dots, h_N\}$ be a finite η-covering subset of $\mathcal{F}^2_{\text{Gauss}}$ under the restricted supremum norm $\|\cdot\|_{\infty,S}$. From Lemma 3, we have $\log N \leq C^* \log m$ for some constant C^*.

Define the index set $J = \{j : \exists h_{0j} \in \mathcal{F}^2_{\text{Gauss}} \text{ s.t. } \|h_{0j} - h_j\|_{\infty,S} \leq \eta \text{ and } \mathcal{D}(h_{0j}, f) \geq t\delta\}$. If $\mathcal{D}(\hat{f}, f) \geq t\delta$, there exists an index $j \in J$ such that $\|\hat{f} - h_{0j}\|_{\infty,S} \leq 2\eta$. Consequently, for all $(\alpha_i, \beta_i) \in S$:

$$\hat{f}(\alpha_i, \beta_i) \leq 2\eta + h_{0j}(\alpha_i, \beta_i).$$

Using the AMLE property of $\hat{f}$ and the Markov inequality:

$$\begin{aligned}
\mathbb{P}_f\left\{\mathcal{D}(\hat{f}, f) \geq t\delta \cap A_t\right\} &\leq \mathbb{P}_f\left(\max_{j\in J} \prod_{i=1}^m \frac{2\eta + h_{0j}(\alpha_i, \beta_i)}{f(\alpha_i, \beta_i)} \geq 1, \forall (\alpha_i, \beta_i) \in S\right) \\
&\leq \sum_{j \in J} \mathbb{E}_f\left[\prod_{i=1}^m \sqrt{\frac{2\eta + h_{0j}(\alpha_i, \beta_i)}{f(\alpha_i, \beta_i)}} \mathbf{1}\{(\alpha_i, \beta_i) \in S\}\right].
\end{aligned}$$

Let D_j denote the expectation term for a fixed j. Since the samples are i.i.d.:

$$D_j = \prod_{i=1}^m \mathbb{E}_f\left[\sqrt{\frac{2\eta + h_{0j}(\alpha_i, \beta_i)}{f(\alpha_i, \beta_i)}} \mathbf{1}\{(\alpha_i, \beta_i) \in S\}\right].$$

Using the inequality $\log x \leq x - 1$:

$$D_j \leq \exp\left(\sum_{i=1}^m \left(\mathbb{E}_f\left[\sqrt{\frac{2\eta + h_{0j}}{f}} \mathbf{1}_S\right] - 1\right)\right).$$

Consider the inner expectation $E := \mathbb{E}_f[\sqrt{(2\eta + h_{0j})/f}\mathbf{1}_S]$. Using $\sqrt{a+b} \leq \sqrt{a} + \sqrt{b}$:

$$\begin{aligned}
E &= \int_S \sqrt{2\eta + h_{0j}(\alpha, \beta)} \sqrt{f(\alpha, \beta)}\, d\alpha d\beta \\
&\leq \int_S \sqrt{2\eta}\sqrt{f}\, d\alpha d\beta + \int_S \sqrt{h_{0j}}\sqrt{f}\, d\alpha d\beta.
\end{aligned}$$

Using Cauchy-Schwarz, $\int_S \sqrt{f} \leq \sqrt{\int_S 1 \cdot \int_S f} \leq \sqrt{|S|}$. Also, recall the Hellinger affinity $\int \sqrt{h_{0j} f} = 1 - \frac{1}{2}h^2(h_{0j}, f)$. Thus:

$$E \leq \sqrt{2\eta}\sqrt{|S|} + 1 - \frac{1}{2}h^2(h_{0j}, f).$$

The volume of S is $|S| \leq 36t^2(M^*)^2$, where $M^* = \max(M_1, M_2, \sqrt{\log m})$. Since $\eta = m^{-2}$, $\sqrt{2\eta} \approx \sqrt{2}/m$. Substituting back into the bound for D_j:

$$D_j \leq \exp\left(m\sqrt{2\eta}\sqrt{|S|} - \frac{m}{2}h^2(h_{0j}, f)\right)$$
$$\leq \exp\left(20.31t^2(M^*)^2 - \frac{m}{2}\mathcal{D}(h_{0j}, f)\right).$$

Since $j \in J$, we have $\mathcal{D}(h_{0j}, f) \geq t^2\delta^2$. (Note: The lemma statement used $t\delta$ for the distance, implying a squared distance in the exponent. Assuming $\mathcal{D}$ is the squared Hellinger distance).
Summing over all $j \in J$:

$$\mathbb{P}_f\left\{\mathcal{D}(\hat{f}, f) \geq t\delta \cap A_t\right\} \leq N \exp\left(20.31t^2(M^*)^2 - \frac{mt^2\delta^2}{2}\right).$$

Combining this with the bound on $\mathbb{P}(A_t^c)$ and using $\log N \leq C^* \log m$, the result follows. □

Lemma 5. *Let $\widehat{G}$ be the EM estimate of the prior G, and let $f_{\widehat{G}}$ and f_G denote the corresponding mixture densities. Under the assumption that $f_{\widehat{G}}$ is an Approximate Maximum Likelihood Estimator (AMLE) of f_G,*

$$W(G, \widehat{G}) \to 0, \tag{S5.25}$$

where $W(\cdot, \cdot)$ denotes the Wasserstein distance.

Proof. For notational convenience, we denote the mixture density determined by a prior G as f_G. Consequently, $\hat{f}$ and $f_{\widehat{G}}$ are used interchangeably. By the AMLE assumption, we have:

$$\prod_{i=1}^{m} \frac{f_{\widehat{G}}(\sqrt{n}\alpha_i, \sqrt{n}\beta_i)}{f_G(\sqrt{n}\alpha_i, \sqrt{n}\beta_i)} \geq 1.$$

We first formalize the Wasserstein distance. Consider two discrete mixing distributions with d components:

$$G = \sum_{i=1}^{d} \pi_i \delta(\boldsymbol{\mu}_i, \boldsymbol{\Sigma}_i), \quad G' = \sum_{i=1}^{d} \pi_i' \delta(\boldsymbol{\mu}_i', \boldsymbol{\Sigma}_i').$$

A coupling between the mixing proportions $\boldsymbol{\pi}$ and $\boldsymbol{\pi}'$ is a matrix $\mathbf{q} = (q_{ij}) \in [0, 1]^{d \times d}$ satisfying the marginal constraints $\sum_{j=1}^{d} q_{ij} = \pi_i$ and $\sum_{i=1}^{d} q_{ij} = \pi_j'$. Let $\mathcal{Q}(\boldsymbol{\pi}, \boldsymbol{\pi}')$ denote the space of all such couplings. The Wasserstein distance between G and G' is defined as:

$$W(G, G') = \inf_{\mathbf{q} \in \mathcal{Q}(\boldsymbol{\pi}, \boldsymbol{\pi}')} \sum_{i,j} q_{ij} \left(\left\|\boldsymbol{\mu}_i - \boldsymbol{\mu}_j'\right\| + \left\|\boldsymbol{\Sigma}_i - \boldsymbol{\Sigma}_j'\right\|\right), \tag{S5.26}$$

where $\|\cdot\|$ denotes the appropriate Euclidean or Frobenius norm.
Established results in mixture model theory provide bounds relating the Wasserstein distance to the Total Variation (TV) distance. Specifically, [5] (elaborated in Example 2.1 of [3]) show that for any two mixing measures G_1 and G_2:

$$TV(f_{G_1}, f_{G_2}) \leq C_1 W(G_1, G_2),$$

where C_1 is a constant depending on the parameter space bounds (c, C, M). Conversely, since $\widehat{G}$ is an AMLE in the exact fitted setting and Gaussian location-scale families

satisfy first-order identifiability (Theorem 3.4 of [3]), Corollary 3.1 of [3] implies the lower bound:

$$TV(f_{\widehat{G}}, f_G) \geq C_0 W(\widehat{G}, G),$$

where C_0 is a constant dependent on G. Furthermore, the relationship between Total Variation and Hellinger distance is given by $TV(f_{\widehat{G}}, f_G) \leq \sqrt{2}\sqrt{\mathcal{D}(f_{\widehat{G}}, f_G)}$. Combining these inequalities with Lemma 4 (which establishes that $\mathcal{D}(f_{\widehat{G}}, f_G) \to 0$), we obtain:

$$C_0 W(\widehat{G}, G) \leq TV(f_{\widehat{G}}, f_G) \leq \sqrt{2}\sqrt{\mathcal{D}(f_{\widehat{G}}, f_G)} \to 0.$$

Thus, $W(\widehat{G}, G) \to 0$ as $m \to \infty$.
Finally, we note that the Wasserstein metric $W(G, G')$ vanishes if and only if G and G' are identical up to a permutation of their atoms (given the non-singularity of the exact fitted setting). Since the EM algorithm preserves this permutation invariance, the convergence $W(\widehat{G}, G) \to 0$ implies the pointwise convergence of the EM estimates to the true parameters (up to permutation). □

Lemma 6. *Let $(a_i, b_i) \sim \mathcal{F}^2_{Gauss}$ for $i = 1, \ldots, m$. Let f (equivalently f_G) and $\widehat{f}$ (equivalently $f_{\widehat{G}}$) be defined as in Lemma 5. Let $lfdr(a, b)$ and $\widehat{lfdr}(a, b)$ denote the local FDRs defined based on f and $\widehat{f}$ respectively, evaluated at (a, b). Under the AMLE assumption, as $m \to \infty$,*

$$\frac{1}{m}\sum_{i=1}^{m}\left|\widehat{lfdr}(a_i, b_i) - lfdr(a_i, b_i)\right| \xrightarrow{P} 0.$$

Proof. We decompose the mean absolute difference as follows:

$$\begin{aligned}
\frac{1}{m}\sum_{i=1}^{m}\left|\mathrm{lfdr}(a_i, b_i) - \widehat{\mathrm{lfdr}}(a_i, b_i)\right| &= \frac{1}{m}\sum_{i=1}^{m}\left|\frac{\pi_{11} f_{11}(a_i, b_i)}{f(a_i, b_i)} - \frac{\hat{\pi}_{11}\hat{f}_{11}(a_i, b_i)}{\hat{f}(a_i, b_i)}\right| \\
&= \frac{1}{m}\sum_{i=1}^{m}\left|\frac{\pi_{11} f_{11}\hat{f} - \hat{\pi}_{11}\hat{f}_{11} f}{f\hat{f}}\right| \\
&\leq I + II,
\end{aligned}$$

where the terms I and II (omitting arguments (a_i, b_i) for brevity) are defined as:

$$\begin{aligned}
I &= \frac{1}{m}\sum_{i=1}^{m}\left|\frac{\pi_{11} f_{11}\hat{f} - \hat{\pi}_{11} f_{11}\hat{f}}{f\hat{f}}\right| = \frac{1}{m}\sum_{i=1}^{m}\frac{f_{11}|\pi_{11} - \hat{\pi}_{11}|}{f}, \\
II &= \frac{1}{m}\sum_{i=1}^{m}\left|\frac{\hat{\pi}_{11} f_{11}\hat{f} - \hat{\pi}_{11}\hat{f}_{11} f}{f\hat{f}}\right|.
\end{aligned}$$

We examine the convergence of these terms separately.
Term I:

$$\begin{aligned}
I &= |\pi_{11} - \hat{\pi}_{11}|\frac{1}{m}\sum_{i=1}^{m}\frac{f_{11}(a_i, b_i)}{f(a_i, b_i)} \\
&\leq \frac{|\pi_{11} - \hat{\pi}_{11}|}{\pi_{11}} \xrightarrow{P} 0 \quad \text{(by Lemma 5).}
\end{aligned}$$

Term II: Applying the triangle inequality, we bound II as:

$$
\begin{aligned}
II &\le \frac{1}{m}\sum_{i=1}^{m}\frac{|\hat{\pi}_{11}|}{f\hat{f}}\left(f_{11}|\hat{f}-f| + f|\hat{f}_{11}-f_{11}|\right) \\
&\le \frac{1}{m\pi_{11}}\sum_{i=1}^{m}\frac{\left|\hat{f}(a_i,b_i)-f(a_i,b_i)\right|}{\hat{f}(a_i,b_i)} + \frac{1}{m}\sum_{i=1}^{m}\frac{\left|\hat{f}_{11}(a_i,b_i)-f_{11}(a_i,b_i)\right|}{\hat{f}(a_i,b_i)} \\
&:= III + IV.
\end{aligned}
$$

To prove the convergence of III and IV, consider the truncation set:

$$
S_m = \left[-M_1 - 2t\sqrt{\log\log m}, M_1 + 2t\sqrt{\log\log m}\right] \times \left[-M_2 - 2t\sqrt{\log\log m}, M_2 + 2t\sqrt{\log\log m}\right],
$$

and the event $A_t = \bigcap_{i=1}^{m}\{(a_i,b_i)\in S_m\}$. Following the arguments in Lemma 4, $\mathbb{P}(A_t^c) \le 2(\log m)^{-t^2/C}$ for some constant $C>0$. Furthermore, for any $(a,b)\in S_m$, the estimated density is bounded away from zero: $\hat{f}(a,b) \ge K_1(\log m)^{-2t^2/C}$.
Convergence of III: For any $\delta > 0$:

$$
\begin{aligned}
\mathbb{P}(III > \delta t) &\le \mathbb{P}(\{III > \delta t\}\cap A_t) + \mathbb{P}(A_t^c) \\
&\le \mathbb{P}(III > \delta t \mid A_t)\mathbb{P}(A_t) + 2(\log m)^{-t^2/C}.
\end{aligned}
$$

Conditioning on A_t, and utilizing the bound on $\hat{f}$:

$$
\mathbb{P}(III > \delta t \mid A_t) \le \mathbb{P}\left(\frac{1}{m}\sum_{i=1}^{m}\left|\hat{f}(a_i,b_i)-f(a_i,b_i)\right| > K\delta t(\log m)^{-2t^2/C} \;\middle|\; A_t\right).
$$

Using the relationship between the L_1 distance, Total Variation (TV), and Hellinger distance ($\mathcal{D}$), we have:

$$
\frac{1}{m}\sum_{i=1}^{m}\left|\hat{f}-f\right| \approx 2TV(f,\hat{f}) \le \sqrt{2}\sqrt{\mathcal{D}(f,\hat{f})}.
$$

Let $t' = K\delta t^2(\log m)^{-4t^2/C}$. Applying the tail bound from Lemma 4:

$$
\begin{aligned}
\mathbb{P}(III > \delta t) &\le \mathbb{P}\left(\mathcal{D}(f,\hat{f}) > \delta t'\right) + \mathbb{P}(A_t^c) \\
&\le C^*\log m \exp\left(K_1 t^4(\log m)^{-\frac{8t^2}{C}}(M^*)^2 - \frac{mK_2 t^4(\log m)^{-\frac{8t^2}{C}}}{2}\right) \\
&\quad + 2m^{-t'^2/C} + 2(\log m)^{-t^2/C}.
\end{aligned}
$$

Using L'Hôpital's rule, it can be verified that for an appropriate choice of t (e.g., $t = \sqrt{C}/4$), the RHS converges to 0 as $m \to \infty$.
Convergence of IV: For $(a,b)\in S_m$, the function $f_{11}(a,b)$ is continuously differentiable with respect to the parameters (μ,θ,κ,ψ). Thus, it is Lipschitz continuous on the bounded domain S_m. There exists a constant C_1 such that:

$$
\left|\hat{f}_{11}(a,b) - f_{11}(a,b)\right| \le C_1\sqrt{(\mu-\hat{\mu})^2 + (\psi-\hat{\psi})^2 + (\theta-\hat{\theta})^2 + (\kappa-\hat{\kappa})^2}.
$$

Let q be the minimum non-zero entry in the optimal coupling matrix $\mathbf{q}$ for the Wasserstein metric $W(G,\hat{G})$. The Euclidean distance between the parameters is bounded by the Wasserstein distance:

$$
\sqrt{(\mu-\hat{\mu})^2 + \cdots + (\kappa-\hat{\kappa})^2} \le \frac{1}{q}W(G,\hat{G}).
$$

From Lemma 5, we know $W(G, \hat{G}) \leq K_6\sqrt{\mathcal{D}(\hat{f}, f)}$. Therefore:

$$\left|\hat{f}_{11} - f_{11}\right| \leq \frac{C_1 K_6}{q}\sqrt{\mathcal{D}(\hat{f}, f)}.$$

The probability bound for IV:

$$\mathbb{P}(IV > \delta t \mid A_t) \leq \mathbb{P}\left(\mathcal{D}(\hat{f}, f) > K^* \delta^2 t^2 (\log m)^{-4t^2/C} \mid A_t\right),$$

shares the same convergence rate as derived for Term III. Consequently, $IV \xrightarrow{P} 0$. Combining the results for I, III, and IV, the lemma is proved. □

Remark E.1. The theoretical guarantees established in this work rely on the Approximate Maximum Likelihood Estimator (AMLE) condition outlined in Assumption **(A1)**. To empirically validate this assumption, we calculated the proportion of simulation runs where the EM algorithm yielded a likelihood ratio of at least 1. This evaluation was conducted over 100 independent replications for each simulation setting.

Simulation Setup	Proportion (Ratio ≥ 1)
Case 1 (Dense)	1.00
Case 1 (Sparse)	1.00
Case 2 (Dense)	1.00
Case 2 (Sparse)	1.00
Binary Outcome	1.00
Composite Alternative	0.98

These results demonstrate that the AMLE assumption is empirically justified and holds consistently across the model configurations considered in this study.

References

1. Hongyuan Cao, Jun Chen, and Xianyang Zhang. Optimal false discovery rate control for large scale multiple testing with auxiliary information. *Annals of Statistics*, 50:807–857, 2022.

2. Nabarun Deb, Sujayam Saha, Adityanand Guntuboyina, and Bodhisattva Sen. Two-component mixture model in the presence of covariates. *Journal of the American Statistical Association*, 117(540):1820–1834, 2022.

3. Nhat Ho and XuanLong Nguyen. On strong identifiability and convergence rates of parameter estimation in finite mixtures. *Electronic Journal of Statistics*, 10:271 – 307, 2016.

4. Michael Naaman. On the tight constant in the multivariate dvoretzky–kiefer–wolfowitz inequality. *Statistics & Probability Letters*, 173:109088, 2021.

5. XuanLong Nguyen. Convergence of latent mixing measures in finite and infinite mixture models. *The Annals of Statistics*, 41:370–400, 2013.
